# German version of subjective methods for measuring activation, mental workload, and attention in control rooms

**DOI:** 10.3389/fpsyg.2026.1698078

**Published:** 2026-04-07

**Authors:** Estefany Rey-Becerra, Patricia Tegtmeier, Matthias Hartwig, Sascha Wischniewski

**Affiliations:** Federal Institute for Occupational Safety and Health (BAuA), Unit Human Factors, Ergonomics, Dortmund, Germany

**Keywords:** mental workload, energetic arousal, tense arousal, flow experience, control rooms

## Abstract

This manuscript focuses on selecting and testing subjective assessment methods to measure mental strain in control rooms. Based on interviews with energy sector workers, our framework includes activation, mental workload, and attention, as they were closely linked to experienced mental stress during critical decision-making situations. Consequently, we translated the Activation–Deactivation Adjective Check List (AD–ACL) and the Workload Profile (WP) into German and validated them alongside the Flow Experience questionnaire. To assess their applicability, 96 volunteers performed two tasks (single-task and multitask) of varying difficulty at different times of day. Exploratory and confirmatory factor analyses supported the internal structure of the AD–ACL and Flow scales, while internal consistency was adequate across all instruments. Convergent and discriminant validity were verified through correlations between self-report measures and task performance. Additionally, all instruments showed sensitivity to task difficulty manipulations. These findings support the use of these tools in human-computer interaction research and their potential for assessing adaptive assistance systems in control rooms.

## 1 Introduction

The energy supply sector faces major workplace challenges. The *Stress Report Germany* (BIBB/BAuA) 2024 reveals that it had the second-highest rise in work-related mental stress among dependent employees in all surveyed industries (Bundesanstalt Für Arbeitsschutz Und Arbeitsmedizin, 2024). While the percentage of employees reporting stress previously declined (Lohmann-Haislah, 2012, 2020; Lohmann-Haislah et al., 2020), this trend has reversed, with around 45% now reporting increased stress over the past 2 years, specially control room operators (or dispatchers).

A control room serves as command center where dispatchers oversee and manage critical processes in power generation and distribution (Schwarz et al., 2010). These roles require multitasking, as they must monitor multiple systems while processing information from various sources (Afzal et al., 2022; Jeschke and Lafrenz, 2015), facing constant pressure from potential disruptions,^1^ which can affect millions of citizens (Petermann et al., 2010). The rise of smart systems has added complexity, making failures potentially harder to identify and accelerate their propagation throughout the network (Afzal et al., 2022). To cope with these demands, dispatchers are equipped with assistance systems designed to support network stability (Tran et al., 2007). Yet, to our knowledge, there is still no standardized methodology for evaluating their effectiveness.

These challenges were the starting point of the research project “Optimized Work Design for Network Control rooms of Critical Infrastructure” funded by the Federal Ministry of Research, Technology and Space of Germany (BMFTR, Grant N° 03SF0694) (Federal Institute For Occupational Safety Health, 2022). The project aims to optimize the *mental stress* in electricity and gas industry control rooms through the implementation of assistance systems and simulators.

Using an interdisciplinary approach, the project analyzes working conditions in real settings, with emphasis on the increasing frequency of time-critical decision-making scenarios. A key work package focuses on the ergonomic analysis of mental strain, where contextual factors and relevant user states are identified in order to develop metrics for a multifactorial assessment. Hence, this manuscript focusses on the selection and validation of subjective assessment methods for measuring *mental strain* in control rooms.

Preliminary semi-structured interviews with six dispatchers at a German energy supply company were conducted to identify user states relevant to mental stress in control-room contexts. The interviews revealed that during typical low-activity periods, dispatchers engage in passive monitoring with low mental activation, resulting in monotony and reduced attention. However, sudden events such as network failures increase multitasking and information overload, which elevate mental activation and may lead to fatigue or a highly emotional state characterized by nervousness or even anger. Overall, information overload, multitasking, and sudden changes from calm to high-pressure situations were described as especially exhausting. These challenges negatively impacting concentration, arousal levels, and emotional well-being. Based on these results, we also decided to include other user states beyond mental workload: activation, attention and emotional state (Schwarz, 2019).

A panel of experts linked each construct to an appropriate measurement instrument for our study: (1) the Workload Profile (WP) by Tsang and Velazquez (1996), (2) the Activation–Deactivation Adjective Checklist (AD–ACL) by Thayer (1986), (3) the Flow-Experience Questionnaire by Bartzik et al. (2021a), and (4) the Self-Assessment Manikin (SAM) by Bradley and Lang (1994), respectively. We found that the WP had not yet been translated into German, the AD–ACL had been translated but focused on personality traits rather than arousal states, and the Flow questionnaire had been developed in German but in a different context (nursing). The SAM was excluded from the current study due to existing validated German translations (Fischer et al., 2002) and its pictorial format.

Hence, the present study aims to translate the WP and AD–ACL and evaluate their psychometric properties alongside the Flow questionnaire for use in German. Additionally, we test their sensitivity to variations in task load and circadian conditions in a controlled, control-room-like environment. The intended application of this work is to provide validated tools for assessing operator states in energy control rooms and to support the design and evaluation of assistance systems that enhance performance, reduce fatigue, and maintain emotional well-being.

## 2 Theoretical background

### 2.1 Mental stress, mental strain, and mental workload: conceptual distinctions

The stress-strain model by Rohmert and Rutenfranz (1975)^2^ has evolved from a research construct to an international standardized framework in the ISO 10075-1 on “Ergonomic principles related to mental workload” (Rohmert and Raab, 1995; Rosen, 2023, p. 28). Although some authors—including Louis et al. (2023)—have linked the stress–strain distinction to later models (e.g., Karasek, 1979), its origin lies in ergonomic theory, specifically in the German tradition of occupational physiology (Ferreira and Vogt, 2022).

The standard defines *mental stress*, as “total of all assessable influences impinging upon a human being from external sources and affecting that person mentally” (Deutsche Institut Für Normung (Din En), 2017). It is a neutral, external factor that varies based on working conditions, such as work tasks, equipment, environmental factors, organizational structure, and workplace characteristics (Joiko et al., 2010; Nachreiner, 1999). Task load—the demands arising from the task itself—is thus considered a key component of mental stress (Nachreiner, 1999).

**Figure 1:**
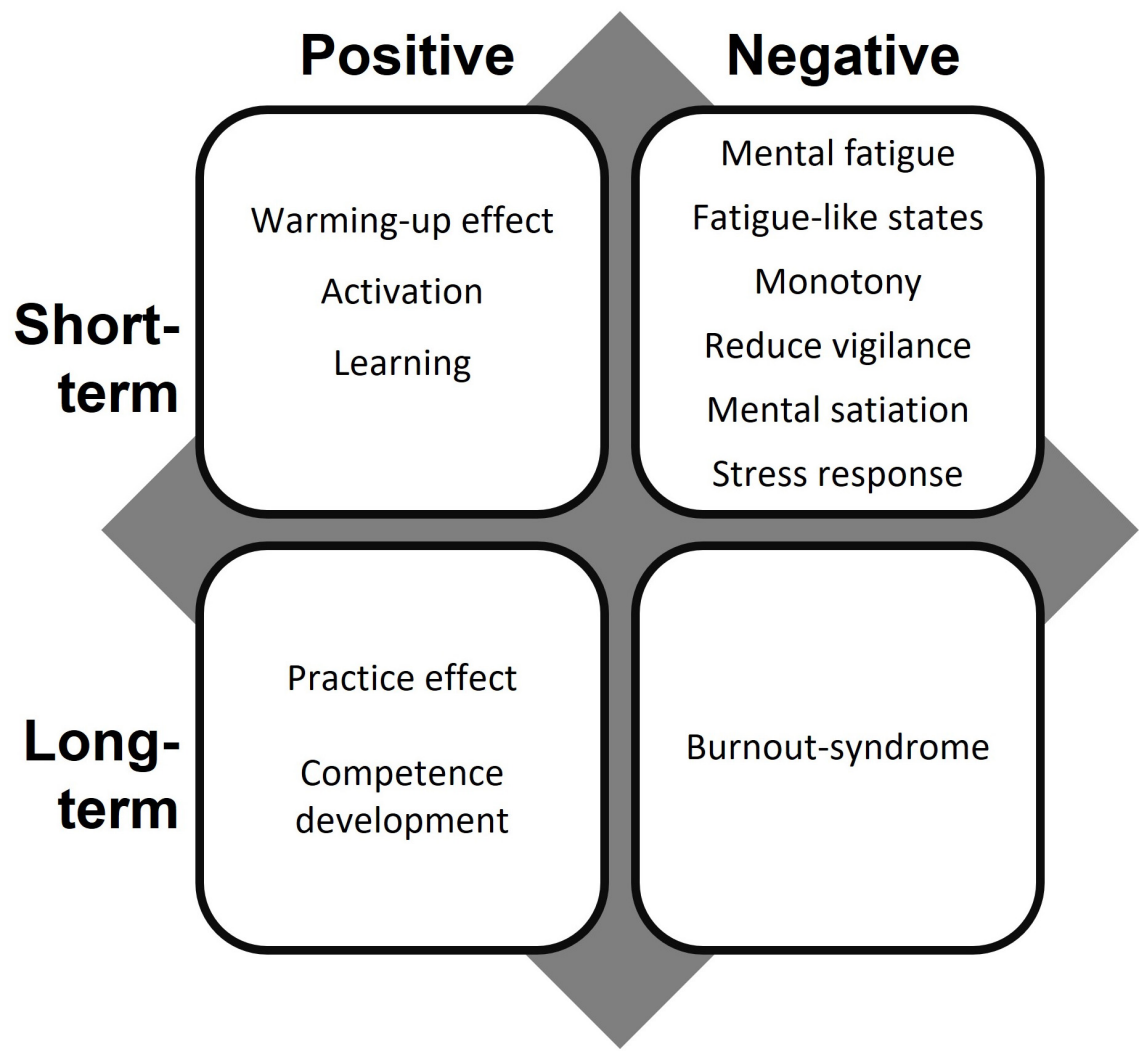
Consequences of mental strain, adapted from DIN EN ISO 10075-1, 2017.

In contrast, *mental strain* is define as “the immediate effect of mental stress on an individual” (DIN ISO 10075-1, 2017). It is a person-specific, internal state that results from the interaction between mental stressors and individual characteristics—such as abilities, demographics, personality, coping mechanisms, self-efficacy, and prior experience—which determine how mental stress impact on mental strain (Semmer, 2002; Sharma and Devi, 2011). This means that two individuals may experience different levels of strain under identical stress conditions. The intensity and duration of mental stress, combined with these factors, determine whether the resulting mental strain is optimal, too low, or too high, leading to positive or negative consequences (Figure 1; Hacker and Richter, 1984).

Cognitive or mental workload—terms that Hancock et al. (2021, p. 204) note are essentially equivalent—is a multidimensional concept encompassing both mental stress and mental strain (Louis et al., 2023). It is often used as an umbrella term for cognitive demands during task performance, including attention, perception, and information processing (Metzler, 2018, p. 6). However, while widely adopted across disciplines, terminological inconsistencies persist (Longo, 2015). Over 60 different definitions of mental workload have been documented in the literature (Longo et al., 2022), leading to frequent misuse of terms. In human factors research and ergonomics, researchers commonly rely on subjective self-report tools to assess what they term cognitive or mental workload, capturing perceived effort and stress-related responses (Tierney et al., 2025; Young et al., 2015), phenomena that, under the ISO 10075-1, would more accurately fall under the definition of “mental strain”.

To bridge these terminological differences, Longo et al. (2022, p. 18) proposed a more comprehensive definition of mental workload: “it represents the *degree of activation* of a *finite resource pool* with limited capacity in the cognitive processing of a primary task over time, which is influenced by external stochastic environmental and situational factors as well as by certain internal characteristics of a person to cope with static task requirements through effort and *attention*”. This definition emphasizes three key components that form the foundation of our assessment approach: activation, finite resource pool, and attention. In the present study, each component is mapped to a specific instrument for measurement: activation is assessed with the AD–ACL, the finite resource pool is captured using the Workload Profile, and attention is evaluated via the Flow Experience questionnaire. These components align conceptually with ISO's notion of mental strain while providing a framework for understanding how control room operators experience mental demands during critical decision-making situations.

### 2.2 Activation and arousal states

In Longo's definition, *activation* reflects the individual's response to stress, acting as a central mechanism in mental workload emergence. It is an “internal state resulting in increased mental and physical activity,” which must stay within an optimal range to maintain performance, reflecting the readiness of individuals to engage with tasks (DIN ISO 10075-1, 2017). Gaillard (1993) established that energetical processes, a generic term for activation, arousal, and fatigue, regulate the organism's state and indirectly influence information processing. This dynamic spans a cognitive load spectrum, where both overload and underload have significant challenges (Debitz et al., 2012).

Assessment methods have consequently focused on establishing thresholds between levels of mental workload: underload, where available resources vastly outstrip demands (e.g., insufficient task load caused by lack of work, overly simple tasks, or underutilized skills) and may result in monotony, in turn causing passive fatigue; and overload, where demands exceed available capacity (e.g., excessive task load caused by too much work, time pressure, or complex tasks) and may lead to active fatigue (Hancock et al., 2021; Matthews and Reinerman-Jones, 2017). It is therefore essential to find a balance that avoids both extremes through moderate workload, where the operator has sufficient resources to meet challenging demands, a state that tends to improve alertness and activation.

In this regard, Thayer (1986) developed the Activation–Deactivation Adjective Checklist (AD–ACL) to measure such activation states based on two orthogonal dimensions: energetic and tense arousal (Thayer, 1978, 1989, 1996). Energetic arousal, ranging from alertness to fatigue, is influenced by multiple factors,^3^ including the sleep-wake cycle and physical activity, and is sensitive to stimulants and circadian rhythms (Košćec and Radošević-Vidaček, 2004; Matchock and Mordkoff, 2009). In contrast, tense arousal, from calmness to tension, is more strongly associated with psychological responses to perceived threat or challenge (Košćec and Radošević-Vidaček, 2001, 2004). These dimensions reflect different affective states: energy and calmness are associated with positive affect (e.g., alertness or enthusiasm, relaxation or serenity), while tension and fatigue represent negative affect (e.g., anxiety or stress, exhaustion or disengagement; Thayer, 1989). Besides, tense arousal is more sensitive to stress-inducing conditions than energetic arousal (Matthews, 2006; Matthews et al., 1990), suggesting that multitasking and high task difficulty might elevate tension.

As an adjective-based questionnaire, the AD–ACL has been translated into multiple languages (e.g., Košćec and Radošević-Vidaček, 2001; Van Dongen and Dinges, 2000). However, literal translations often overlook contextual nuances. Even within English dialects, semantic equivalence (Mackay et al., 1978; Mccormick et al., 1985), and factor structures (Fischer and Donatelli, 1987) varies across countries. Moreover, language evolves and some adjectives become outdated or shift in meaning (Altakhaineh, 2018), underscoring the need for cultural adaptation. For these reasons, we have decided to retranslate the AD–ACL despite the existence of two German versions, including one by Imhof (1998), based on Wieland-Eckelmann and Bösel (1987). These earlier translation by Wieland-Eckelmann and Bösel (1987) primarily focused on personality traits such as trait anxiety rather than arousal states. The existing German version by Imhof (1998) did not yield a clear factor solution for at least one item (in German: *jittery*), for which the author herself proposed an alternative translation. Several items also showed semantic shifts since the mid-1980s or lacked contemporary associations with activeness (e.g., in German: *frisch*). Without systematic comparisons in the original publications, it remained unclear which items required adaptation. Taken together, these psychometric and semantic limitations motivated a complete retranslation of the instrument. We therefore hope that this new German version of the AD–ACL will better reflect current conceptions of energetic and tense arousal and reveal variations in both dimensions depending on the task conditions. Consequently, our hypotheses are:

**Hypothesis 1**. The energetic arousal of the AD–ACL German version will vary depending on the time of day before and after the task, reflecting circadian effects seen in the original version.

**Hypothesis 2**. The tense arousal of the AD–ACL German version will vary depending on the task condition and difficulty level after completing the task.

### 2.3 Finite resource pool and mental workload assessment

The International Labor Organization (ILO, 2011) outlines two main approaches to understanding mental workload: the task-requirements approach (Hancock and Chignell, 1986; Welford, 1978), which views workload as external demands imposed by tasks, and the resource-interaction approach (Gopher and Donchin, 1986), which defines it as the interaction between task demands and individual cognitive and emotional resources. While the former focuses on task characteristics, the latter emphasizes subjective experience and explains individual differences in coping with workload. This resource-interaction approach is exemplified in Longo's definition of workload, which specifically mentions a “*finite resource pool*” explained by the Wickens' *Multiple Resource Theory* (MRT). According to (Wickens 1992, 2008), various cognitive resources process mental stress, and performance declines when tasks compete for the same resource. Those resources are classified according to the information processing (perception, central processing, action execution), modality (visual, auditory), encoding (verbal, spatial), and visual channel (focal, peripheral). Task interference increases when tasks share similar attributes, explaining multitasking effects (Wickens, 2020), including those specific to the energy sector (Afzal et al., 2022).

There are several psychometric tools to measure mental workload, such as the well-known NASA Task Load Index (TLX; Hart, 2006; Hart and Staveland, 1988), the Subjective Workload Assessment Technique (SWAT; Reid and Nygren, 1988) or the Workload Profile (WP; Tsang and Velazquez, 1996). Although WP is not the most common instrument (Longo et al., 2022), it has shown greater sensitivity to task variations (Barajas-Bustillos et al., 2023; Rubio et al., 2004), attributable to its MRT basis (Tsang and Velazquez, 1996). Unlike the NASA-TLX, which provides a global and partly overlapping workload score, the WP explicitly assesses the consumption of distinct cognitive resources postulated by the MRT by means of separate items for each resource dimension. This allows a fine-grained characterization of mental workload, particularly with respect to multitasking effects.

There have been some applications of this instrument in control room contexts or in other languages. In a recent investigation in Australia, the English WP was used to assess mental workload in the energy sector (Afzal et al., 2022). In another study, the Spanish WP was applied to integrate workload and human error analysis in product design (Durán-Coronado et al., 2019). However, to our knowledge, the WP has not been translated or validated in German. Hence, a German WP is needed for accurate mental workload assessment in high-demand, multitasking contexts like control rooms. Based on the MRT, we expect to observe significantly different resource utilization profiles depending on task difficulty and multitasking demands, leading to the following hypotheses:

**Hypothesis 3**. In the German version of the WP, estimates for all resources in multitasking conditions will be higher than for single-task conditions.

**Hypothesis 4**. In the German version of the WP, estimates for all resources used in difficult tasks will be higher than for non-difficult tasks.

### 2.4 Attention and flow experience

Mental workload is closely tied to the energetic aspects of attention. Wickens (2020) describes attention as both a selective filter and a provider of cognitive resources essential for working memory. When task demands surpass available cognitive resources, performance may decline due to cognitive overload (Cowan, 2001, 2008), especially in multitasking contexts requiring sustained attention (Wickens et al., 2022). As humans have inherent limitations in the processing of simultaneous stimuli (Cowan, 2001), we distribute attentional resources across the various dimensions of the task (Wickens, 1992, 2008). In general, attention itself represents a mental state characterized by heightened alertness and receptiveness, where consciousness focuses on specific information (Schaub, 2008).

Stimuli perceived consciously or unconsciously can become an “attention burden” (Joiko et al., 2010). When personal capabilities align with task demands, individuals may experience *flow*—a state of deep concentration and control under stress, perceived as pleasant and rewarding, with clear feedback and a clear goal (Csikszentmihalyi, 1975; Peifer, 2017). Flow experience has been negative associated with burnout symptoms (Aust et al., 2022), suggesting that flow may serve as a protective factor against burnout by transforming acute stress into pleasant challenges through cognitive reappraisal (Peifer and Wolters, 2021). Besides, research has shown that multitasking disrupts flow by dividing attention and raising cognitive load (Peifer and Zipp, 2019; Pluut et al., 2024), making flow rare when tasks are too hard (Baumann and Scheffer, 2010); nevertheless, it is most common when task difficulty matches individual's skill level (Peifer and Zipp, 2019).

In demanding work settings, Bartzik et al. (2021a) developed a questionnaire to capture the experience of flow at work in terms of absorption, perceived demand-skill balance, and enjoyment. While this instrument was validated with nursing staff during the Covid-19 pandemic, it requires further validation in complex multitasking monitor-based environments like energy sector control rooms. Hence, we anticipate that the effects of varying task difficulty and multitasking demands will be observable in control room-like tasks. Therefore, we hypothesize that:

**Hypothesis 5**. The experience of flow will occur less frequently in a multitasking condition than in a single-task condition.

**Hypothesis 6**. The experience of flow will occur less frequently at the high difficulty level than at the low difficulty level.

### 2.5 Performance implications

(Wickens 1992, 2008) also suggests that performance deteriorates when multiple tasks compete for the same cognitive resources. When the combined demand exceeds an individual's capacity, cognitive overload occurs (Cowan, 2001, 2008), impairing performance (Wickens et al., 2022). This is evident in dispatcher roles, where individuals must simultaneously monitor systems, respond to alarms, calls, deal with complex tasks and decision-making under pressure, as they expressed in the interviews. This multitasking and its complexity negatively affect performance (Adler and Benbunan-Fich, 2015; Laarni, 2021), likely due to limited capacity to process multiple stimuli and large amounts of information (Hwang and Jeong, 2018; Jeong and Hwang, 2016). Hence, we propose the following Hypotheses:

**Hypothesis 7**. The performance will be lower in a multitasking condition than in a single-task condition.

**Hypothesis 8**. The performance will be lower at the high difficulty level than at the low difficulty level.

### 2.6 Conceptual framework for measurement validation

In the context of subjective measures of mental workload and operator state, validation is typically understood as an accumulation of evidence from multiple sources, including internal structure, internal consistency, and relations to other variables. In the present study, we do not aim to provide full validation of the instruments, but rather to contribute targeted validity evidence relevant to their use in control-room-like settings.

The hypotheses formulated in the previous sections are therefore in-tended as tests of construct-related and experimental validity. Specifically, they examine whether the translated instruments respond in theoretically expected ways to controlled variations in task load, task difficulty, and time of day. Support for these hypotheses would indicate that the measures are sensitive to known determinants of activation, mental workload, and attention, while systematic deviations from the expected patterns would highlight limitations in the instruments' ability to capture these constructs in the present context.

## 3 Methods

### 3.1 Study design and procedure

**Figure 2:**
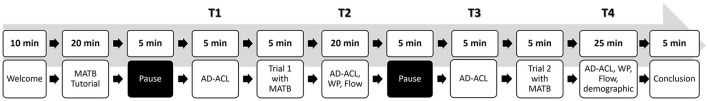
Procedure for the trial study on subjective measurement methods of cognitive stress in control rooms.

This study employed a randomized 3 × 2 × 2 mixed design. Three groups were tested at 10:00, 13:00, and 16:00 (between-subjects) to examine the circadian effect. Two levels of difficulty (between-subjects) and two task conditions (within-subjects: multitasking vs. single-task) were used to assess the effect of tasks complexity. Level of difficulty, task conditions, and group allocations were permuted. The study was conducted in a controlled laboratory environment, with each session lasting approximately 2 h per participant. The experimental procedure is illustrated in Figure 2.

Participants completed control room-like scenarios using the Multi-Attribute Task Battery (MATB; see section 3.3) under varying task load conditions to assess the sensitivity of the German translations. We acknowledge that while MATB provides a simulation of control room tasks with high experimental control, it offers limited ecological validity. Besides, professional control room operators were not recruited for this initial validation study. However, the translated questionnaires have since been then used in real operational settings (Rey-Becerra et al., 2025).

After an introduction, participants received a unique ID-code to ensure anonymized tracking (see section 3.3). They completed the MATB tutorial with an intermedia level of difficulty, followed by two sessions with questionnaire assessments before and after each trial with MATB. The questionnaires were completed via the web-based platform EUSurvey of the Directorate-General for Informatics (DG DIGIT) of the European Commission (2013) using the same computer to test the MATB software. At the end of the study, participants provided sociodemographic information.

### 3.2 Sample size and participants

**Figure 3:**
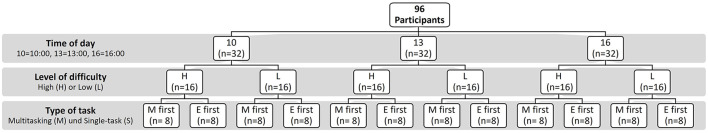
Design of the experiment with the number of persons per group.

*A priori* power analysis using G^*^Power (Faul et al., 2007) determined a required sample of 90 participants to detect medium effects (α = 0.05, 1 – β = 0.95, *f* = 0.25) in two measurements (multitasking vs. single task) and six groups (two difficulty levels × three groups per hour) assuming sphericity correction (ϵ = 1) and a correlation between repeated measures of *r* = 0.5. To align with the factorial design, we considered 96 participants, balanced among conditions as illustrated in Figure 3, with an equal number of male and female participants in each condition.

Participants were recruited via subject pool of the German Federal Institute for Occupational Safety and Health (BAuA), online ads, and flyers. Inclusion criteria included current or prior experience working with computer screens, strong German proficiency, and basic English comprehension. Participants registered via phone or email, and appointments were scheduled at the BAuA technical laboratory in Dortmund, Germany. Upon arrival, participants were informed about the study and signed informed consent forms. Ethical approval was granted by the Ethics Committee of BAuA. Participants received €30 compensation and could withdraw at any time without consequences.

### 3.3 Software and experiment settings

**Figure 4:**
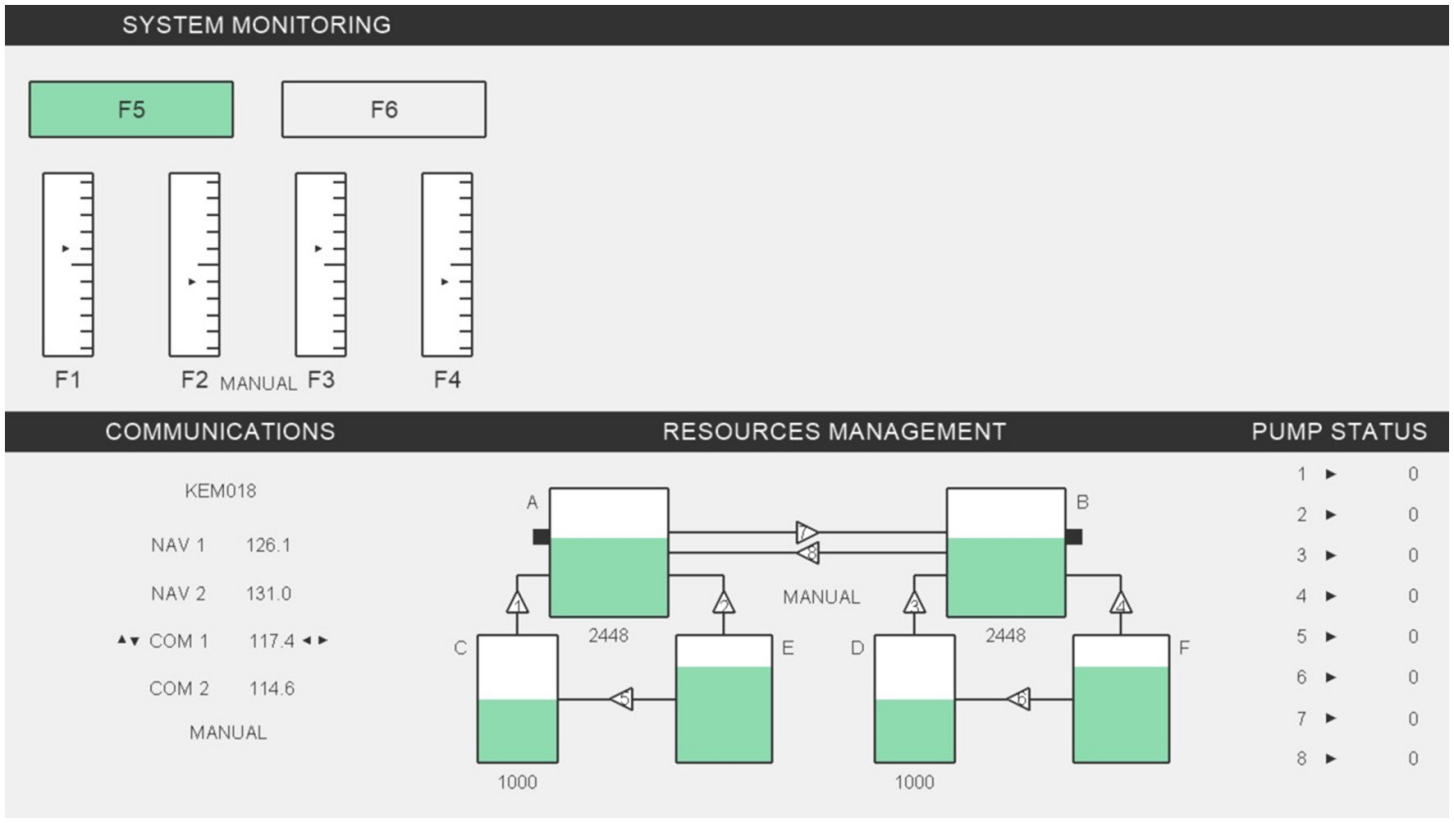
Screenshot of the three MATB tasks used.

Task performance was assessed using a modified version of the Multi-Attribute Task Battery-II (MATB-II; Cegarra et al., 2020; Comstock et al., 1992; Santiago-Espada et al., 2011), which simulates cognitive workload and multitasking in high-demand environments. For this study, three core tasks were implemented as they are particularly relevant to the role of dispatchers: system monitoring (SysMon), communication (Comm), and resource management (ResMan; Figure 4). A detailed description is provided in the Supplementary Appendix A.

Participants used standardized lab equipment (24^′′^ monitor, keyboard, and mouse). They were randomly assigned to one of two difficulty level conditions, and they performed two trials in randomized order: one with a single task (just the ResMan task) and one multitask (with all three tasks simultaneously). In both, only the ResMan task was used for performance assessment. The order of single- and multitask trials was counterbalanced within each difficulty group using a code system. Each code contained a random number and two letters indicating the difficulty level (first letter: N for easy, H for difficult) and trial type (second letter: E for single-task, M for multitask). ID-Codes were distributed equally across three session times (10:00, 13:00, and 16:00), and each participant received one code upon arrival. This procedure ensured an equal distribution of task orders within each difficulty group.

### 3.4 Outcome measurements

We assessed activation and tension with the Activation–Deactivation Adjective CheckList (AD–ACL) developed by Thayer (1986) which contains 20 adjectives capturing two dimensions: energetic arousal and tense arousal. We translate the original version with a multi-stage approach. First, a native German speaker fluent in English generated initial translations using DeepL Write (DeepL SE., 2023) and verified using online dictionaries. For each adjective, three German equivalents were shortlisted and evaluated by nine German experts in human factors and ergonomics, fluent in English. One translation per item was selected by majority vote. Back-translation by a native English speaker fluent in German confirmed fidelity to the original adjectives, with allowances made for synonym shifts. Some of the original adjectives (e.g., “full of pep”) are used less today than when the AD–ACL was created, so they were adapted not only to the language but also to the context (in this case “*voller Elan*”). An initial pilot with 54 participants showed good factorial alignment for energetic arousal, while three adjectives in the tense arousal cluster were revised (quiet, fearful, jittery; in German *still, ängstlich, hibbelig*). The revised terms (*gefasst, unruhig, nervös*) were consistently assigned to the tense arousal dimension by seven additional raters in a blind sorting task. In the current study we used this final version, provided in the Supplementary Appendix B. Since Thayer (1986) pointed out that the type of rating scale has no influence on the factor structure, we used a five-point ranging scale from 1 (not at all—German: *gar nicht*) to 5 (completely—German: *voll und ganz*), labeling only the endpoints. We estimated the activation and tension with the average score of the items included in each factor.

Mental workload was measured using the Workload Profile (WP) questionnaire by Tsang and Velazquez (1996). It includes eight items corresponding to separate cognitive resources (e.g., visual processing, response selection), grounded in Wickens' multiple resource theory, each accompanied by a short explanation. The items were translated into German We translate the original version with a multi-stage approach. Two native German speakers fluent in English independently translated each item and explanation. Some expressions were modified, for instance in *visual information* the word “television” was adapted to “watching videos” to reflect contemporary viewing habits. All items were then back-translated using DeepL Write (Deepl, 2023) and reviewed by a native English speaker fluent in German to ensure semantic accuracy. This version is provided in the Supplementary Appendix B. With that, participants rated perceived resource demand with a continuous scale from 0 to 100, adapted from the original 0–1 range to improve clarity while maintaining proportional meaning. Mean values were computed across items.

Flow experience was assessed using the questionnaire from Bartzik et al. (2021a), available in German (Bartzik et al., 2021b) with 10 items. We used the frequency scale ranging from 1 (never—German: *nie*) to 6 (always—German: *immer/fast immer*), and calculated the average flow experience based on the first nine items.

In addition to the user's states, we assessed participants' performance on the ResMan task of the MATB, following Fairclough et al. (2005). Participants were required to maintain 2500 units of fuel in tanks A and B as it depleted, and performance was measured by calculating the deviation from this level using the root mean squared error at 60 seconds intervals and then averaged, with higher deviations representing lower performance. Finally, a sociodemographic questionnaire was also administered, collecting gender, age, screen usage, consumption of coffee, tea and nicotine, and vision conditions.

### 3.5 Data analysis

Data was extracted from two sources. The first was the computer where participants performed the tasks, generating CSV files with raw data for each trial run in MATB. This data was transformed into performance metrics using an executable file. The second source was from the *EUSurvey* platform (v1.5.3; European Commission, 2013) where participants completed questionnaires, with responses downloaded as Microsoft Excel files. All data preparation, cleaning, and analysis were conducted in RStudio (v4.2.3; R Core Team, 2023).

Exploratory Factor Analysis (EFA) was performed on the German AD–ACL using the *GPArotation* package (v2024.3-1; Bernaards and Jennrich, 2005) with *promax* rotation and a two-factor solution. A loading threshold of ≥0.30 indicated acceptable item-factor association (Hoyle, 2012). Confirmatory factor analyses (CFA) were conducted to evaluate the factorial validity of the German AD–ACL and the Flow scale using the *lavaan* package (v0.6-17, Rosseel, 2012). Given the ordinal response formats, models were estimated using the weighted least squares mean and variance adjusted (WLSMV) estimator. Model fit was assessed using the comparative fit index (CFI), Tucker–Lewis index (TLI), root mean square error of approximation (RMSEA), and standardized root mean square residual (SRMR). Following established guidelines (Hu and Bentler, 1999), CFI and TLI values ≥0.90 indicated acceptable fit and ≥0.95 indicated good fit; RMSEA values ≤ 0.08 indicated acceptable fit and ≤ 0.06 indicated good fit; and SRMR values ≤ 0.08 indicated good fit.

Reliability for all scales was assessed using the *sjPlot* package (v2.8.16; Lüdecke, 2024), with Cronbach's α ≥ 0.70 considered acceptable internal consistency (Tavakol and Dennick, 2011). Descriptive statistics and correlation analyses between constructs were performed using the *psych* package (v2.2.5; Revelle, 2024). These correlations provided evidence of convergent validity (relatedness between theoretically similar constructs) and discriminant validity (distinctness between theoretically different constructs; Kline, 2015). Following Cohen (2013) guidelines, correlation coefficients of 0.1 were considered small, 0.3 moderate, and 0.5 large. We did not exclude outliers, since they are extreme true values (Burke, 1998).

Linear mixed-effects models (LMMs) were used to examine the effects of time of day, task difficulty, and task type on user states and performance. Models were fitted using the lme4 package (v1.1-35; Bates et al., 2015), with participant ID included as a random intercept to account for repeated measurements. Fixed effects were evaluated using Type III ANOVA tables with Satterthwaite-approximated degrees of freedom as implemented in the lmerTest package (v3.1-3; Kuznetsova et al., 2017). Statistical assumptions were confirmed with a significance level set to *p* < 0.05.

*Post-hoc* pairwise comparisons were conducted using the Holm-Bonferroni step-down procedure with the *emmeans* package (v1.10.1; Lenth, 2024) to control the familywise error rate (FWER).^4^ Effect sizes for mixed-effects models were quantified using marginal and conditional *R*^2^ values calculated with the *performance* package (v0.12.0, Lüdecke, 2021). In addition, partial eta-squared ($\eta_{p}^{2}$) values for main effects and interactions were computed using the *effectsize* package (v0.8.7, Ben-Shachar et al., 2020). Following Cohen's (2013) guidelines, values of 0.01 indicate small effects, around 0.06 medium effect, and above 0.14 large effect.

## 4 Results

The study successfully recruited the required 96 participants, with a balanced gender distribution. The average age of the participants was 34.7 years (SD = 12.3). All answered to the questionnaire. While participants stated to had relevant computer experience, they were not professional dispatchers, which should be considered when interpreting the generalizability of our findings to real control-room operators.

Below we show the results of the validation of the translated questionnaires. In addition, we present the main results of the three-way mixed ANOVA to examinate the impact of the MATB, considering the level of difficulty and the condition of the task, for the whole sample (divided into the 3 h of the day), on performance, workload, activation and tension, and the experience of flow.

### 4.1 Activation and tension

We performed an exploratory factor analysis (EFA) on the German translation of the AD–ACL, organized according to Thayer (1986) two core dimensions: The energetic arousal (activation—German: *Aktivierung*), which comprises items from both the energy and tiredness subscales, and the tense arousal (tension—German: *Anspannung*), with items from both tension and calmness. As shown in Table 1, the analysis revealed a clear distinction between the two factors, accounting for 54% of the total variance (28% for activation and 26% for tension), with a moderate negative correlation (*r* = −0.28). Model fit indices indicated an adequate solution (RMSR = 0.06; TLI = 0.815), supporting the bidimensional structure and construct validity of the German AD–ACL.

**Table 1:** Exploratory factor analysis of the AD–ACL: standardized factor loadings.

Subscale	Original item	German item	Activation	Tension	h2	u2	com
Energetic	active	aktiv	0.85		0.68	0.32	1.00
	energetic	tatkräftig	0.80		0.65	0.35	1.00
	full of pep	voller Elan	0.73		0.54	0.46	1.00
	lively	lebhaft	0.73		0.56	0.44	1.00
	vigorous	kraftvoll	0.78		0.66	0.34	1.00
Tired	wide awake	hellwach	0.87		0.71	0.29	1.10
	drowsy^*^	dösig^*^	0.54		0.29	0.71	1.00
	tired^*^	müde^*^	0.68		0.46	0.54	1.00
	wakeful	munter	0.70		0.62	0.38	1.20
	sleepy^*^	schläfrig^*^	0.66		0.41	0.59	1.00
Calmness	at rest^*^	entspannt^*^		0.77	0.58	0.42	1.00
	calm^*^	gelassen^*^		0.75	0.60	0.40	1.00
	quiet^*^	gefasst^*^		0.39	0.22	0.78	1.40
	still^*^	ruhig^*^		0.78	0.61	0.39	1.00
	placid^*^	friedlich^*^		0.61	0.45	0.55	1.10
Tension	jittery	nervös		0.76	0.55	0.45	1.00
	intense	angestrengt		0.75	0.53	0.47	1.10
	tense	angespannt		0.76	0.54	0.46	1.10
	fearful	unruhig		0.80	0.63	0.37	1.00
	clutched up	verkrampft		0.71	0.50	0.50	1.00
		SS loadings	5.56	5.21			
		Proportion Var	0.28	0.26			
		Proportion Explained	0.52	0.48			

Factor loadings ≥0.30 are reported. Items with ^*^ were reversed.

Subsequently, we conducted a confirmatory factor analysis (CFA) to examine the two-dimensional structure of the AD–ACL, specifying two latent factors: activation and tension, in accordance with the results of the previous EFA. The two-factor model showed a mixed fit to the data, CFI = 0.97, TLI = 0.97, RMSEA = 0.14, 90% CI (0.13, 0.14), SRMR = 0.10, indicating good incremental fit, although with high error indices. All standardized factor loadings were statistically significant (*p* < 0.001) and of moderate to strong magnitude, as present in Table 2. The factors were negatively and moderately correlated (*r* = −0.30), consistent with the EFA findings.

**Table 2:** Confirmatory factor analysis of the AD–ACL: two-factors model.

Scale	Original item	German item	Activation	Tension
Energetic	active	aktiv	0.837	
	energetic	tatkräftig	0.862	
	full of pep	voller Elan	0.790	
	lively	lebhaft	0.792	
	vigorous	kraftvoll	0.857	
Tired	wide awake	hellwach	0.824	
	drowsy^*^	dösig^*^	0.659	
	tired^*^	müde^*^	0.778	
	wakeful	munter	0.834	
	sleepy^*^	schläfrig^*^	0.761	
Calmness	at rest^*^	entspannt^*^		0.797
	calm^*^	gelassen^*^		0.833
	quiet^*^	gefasst^*^		0.551
	still^*^	ruhig^*^		0.819
	placid^*^	friedlich^*^		0.776
Tension	jittery	nervös		0.806
	intense	angestrengt		0.752
	tense	angespannt		0.760
	fearful	unruhig		0.851
	clutched up	verkrampft		0.778

All loadings significant (*p* < 0.001). Items with ^*^ were reversed.

We calculated Cronbach's alpha coefficients at four measurement points to assess reliability. Both activation (α = 0.92 at T1, 0.92 at T2, 0.93 at T3, and 0.92 at T4) and tension dimensions (α = 0.88 at T1, 0.90 at T2, 0.90 at T3, and 0.93 at T4) showed excellent internal consistency. The mean inter-item correlations ranged from 0.52 to 0.58 for activation and from 0.43 to 0.56 for tension across measurement points, indicating adequate item homogeneity without redundancy. These results suggest that the German translation maintains a reliability comparable to the original English version.

**Figure 5:**
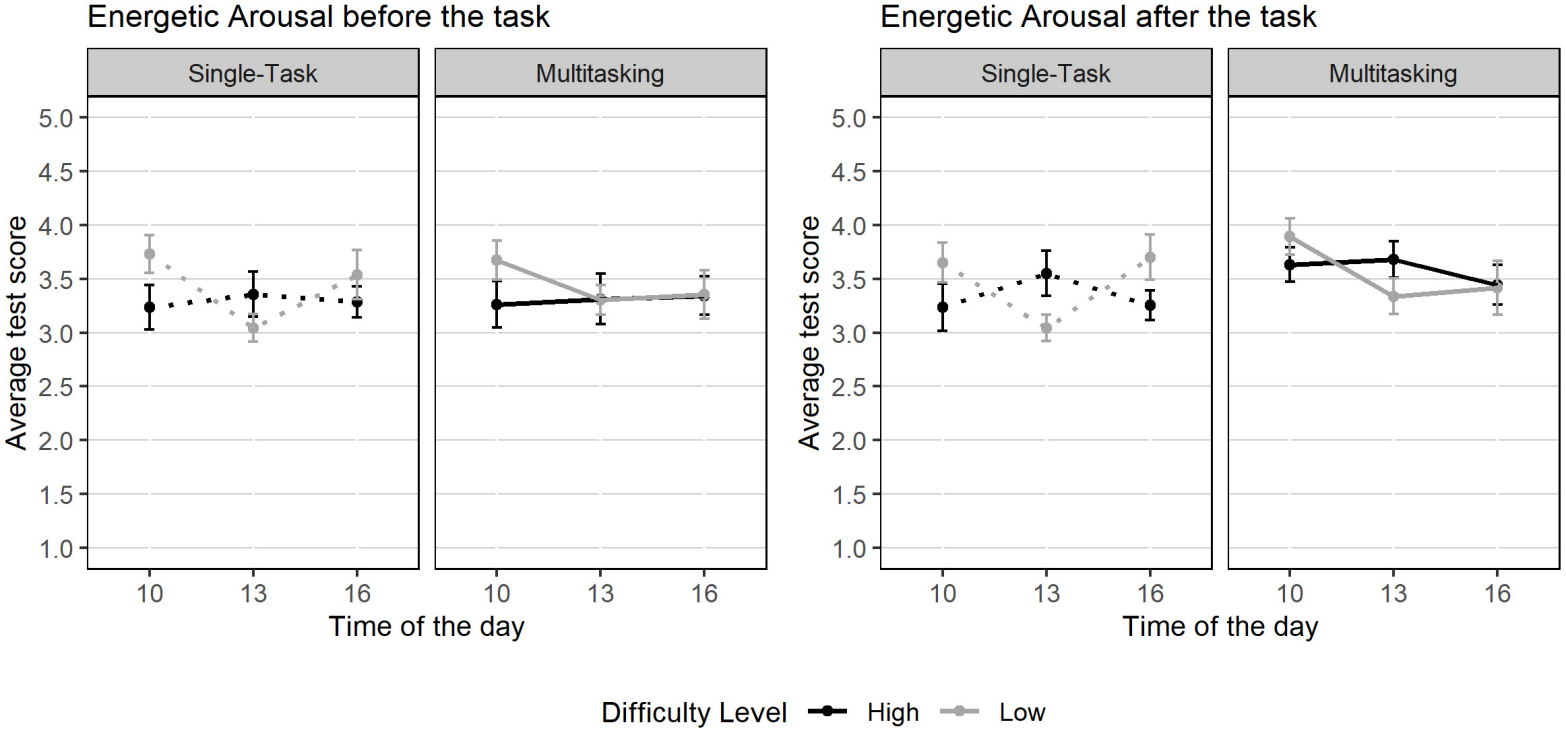
Mean energetic arousal scores before and after the task, by time of day, difficulty, and task condition. Error bars represent the standard error of the mean.

The average energetic arousal values per group range from 3 to 4, which means that there was moderate activation, as shown in Figure 5. Regarding our first hypothesis, we performed a linear mixed-effects model (LMM) with *task condition (multitasking, single-task), difficulty (high, low), group per hour* (10:00, 13:00, and 16:00) and *assessment point* (T1, T2, T3, and T4) as fixed effects and participant as a random intercept (conditional *R*^2^ =0.74, marginal *R*^2^ = 0.14). Results revealed significant main effects for task condition [*F*(1, 252) = 4.10, *p* =0.044, $\eta_{p}^{2}$ = 0.02] and assessment point [*F*(3, 252) = 3.42, *p* =0.018, $\eta_{p}^{2}$ = 0.04]. These were qualified by a significant three-way interaction between group per hour, task condition, and assessment point [*F*(6, 164.9) = 2.34, *p* =0.034, $\eta_{p}^{2}$ = 0.08]. *Post-hoc* planned contrasts compared activation levels before (T1, T3) and after (T2, T4) the tasks. Results indicated a significant increase in activation following the multitasking condition in the 10:00 group (*b* = −0.29, SE = 0.10, *p* =0.005) and a marginal increase in the 13:00 group (*b* = −0.20, SE = 0.10, *p* =0.055), while no changes were observed in the 16:00 group.

**Figure 6:**
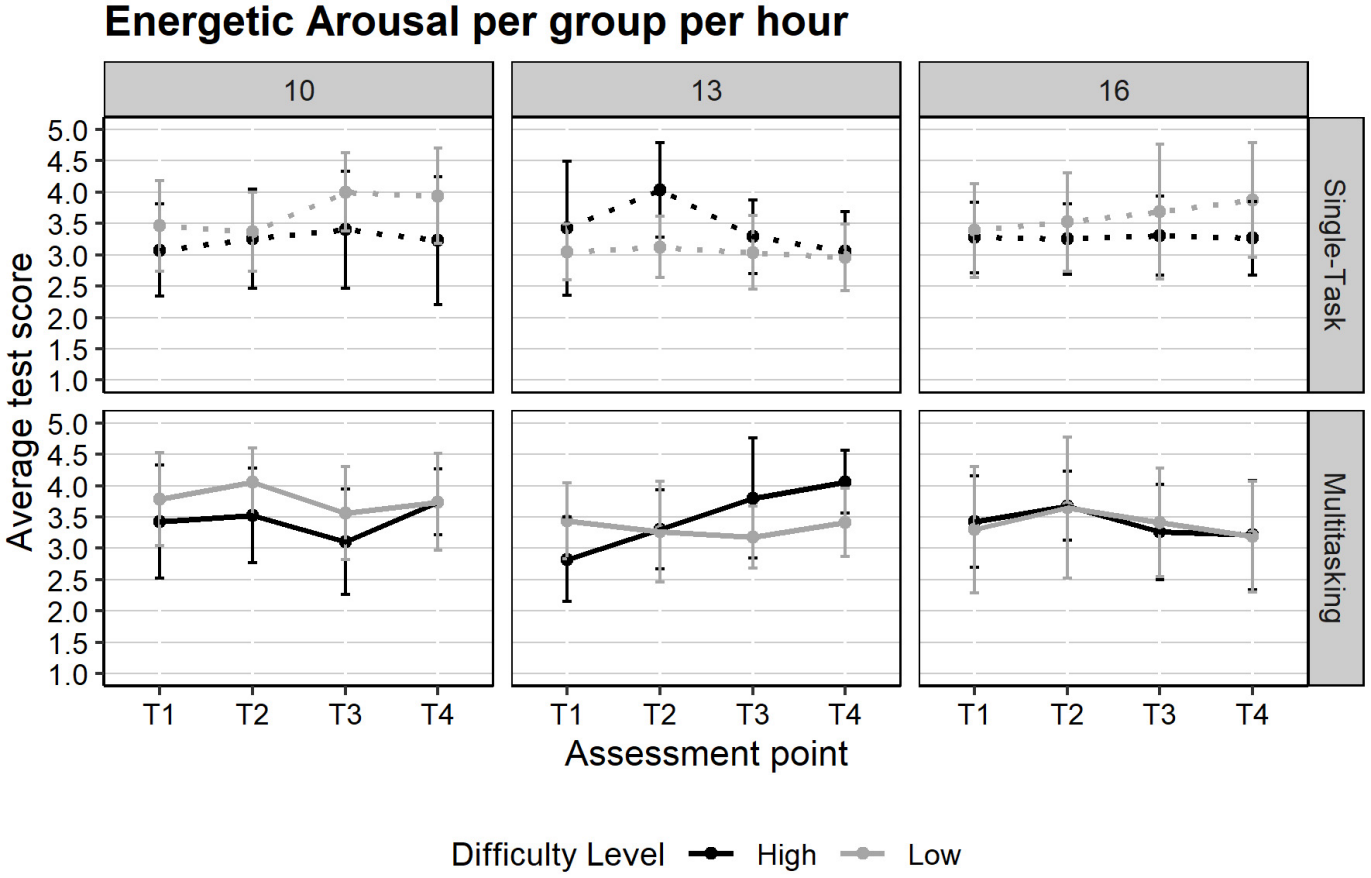
Energetic Arousal across Assessment Points (T1–T4), by time of day, difficulty level, and task condition. Error bars represent the standard error of the mean.

The model also revealed a significant three-way interaction between group per hour, difficulty, and task condition [*F*(2,252) = 4.05, *p* =0.019, $\eta_{p}^{2}$ = 0.03]. As illustrated in Figure 6, participants tested at 13:00 exhibited increased activation levels when performing difficult multitasking, while those at 16:00 showed higher activation during easier single-task conditions. These results suggest that circadian effects influence arousal regulation differently depending on task demands, leading us to partially support **Hypothesis 1**: while activation varied before and after the task as a function of time of day, this circadian effect was only evident under conditions of higher task demand (multitasking).

Concerning tense arousal and our second hypothesis, we performed a LMM with *task condition, difficulty, group per hour* and *assessment point (T1, T2, T3, and T4)* as fixed effects, and participant as a random intercept (conditional *R*^2^ = 0.66, marginal *R*^2^ = 0.21). Results revealed a strong main effect of task condition [*F*(1,276) = 45.33, *p* < 0.001, $\eta_{p}^{2}$ = 0.14], indicating significantly higher tense arousal during multitasking compared to single-task conditions. A significant main effect of assessment point was also observed [*F*(3,276) = 19.99, *p* < 0.001, $\eta_{p}^{2}$ = 0.18], showing systematic changes in tension across the experimental session. These effects were qualified by a significant three-way interaction between task difficulty, task condition, and assessment point [*F*(3,189.9) = 5.60, *p* = 0.001, $\eta_{p}^{2}$ = 0.08]. This indicates that the impact of task difficulty on tense arousal is not uniform but depends on both the nature of the task and the phase of the experiment.

**Figure 7:**
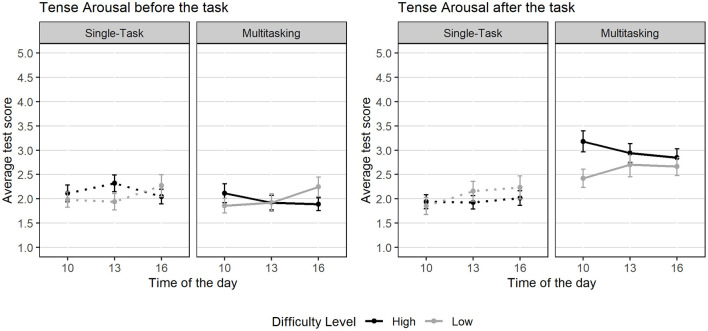
Mean tension scores before and after the task, by time of day, difficulty level, and task condition. Error bars represent the standard error of the mean.

After the task, the task condition effect became highly significant, showing a substantial difference in tension between single-task and multitasking conditions, as shown in Figure 7. *Post-hoc* comparisons confirmed that at the first assessment after the task (T2), multitasking with high difficulty elicited the highest levels of tension (b = 0.67, SE = 0.21, *p* = 0.007). Interestingly, this difficulty-driven gap vanished by the end of the second trial (T4), where multitasking increased tension to a high level regardless of difficulty (*p* = 0.80). Overall, these results support **Hypothesis 2**: tension levels after the task depend on an interaction between task condition and difficulty level, particularly during the initial phase of the experiment.

### 4.2 Mental workload

**Figure 8:**
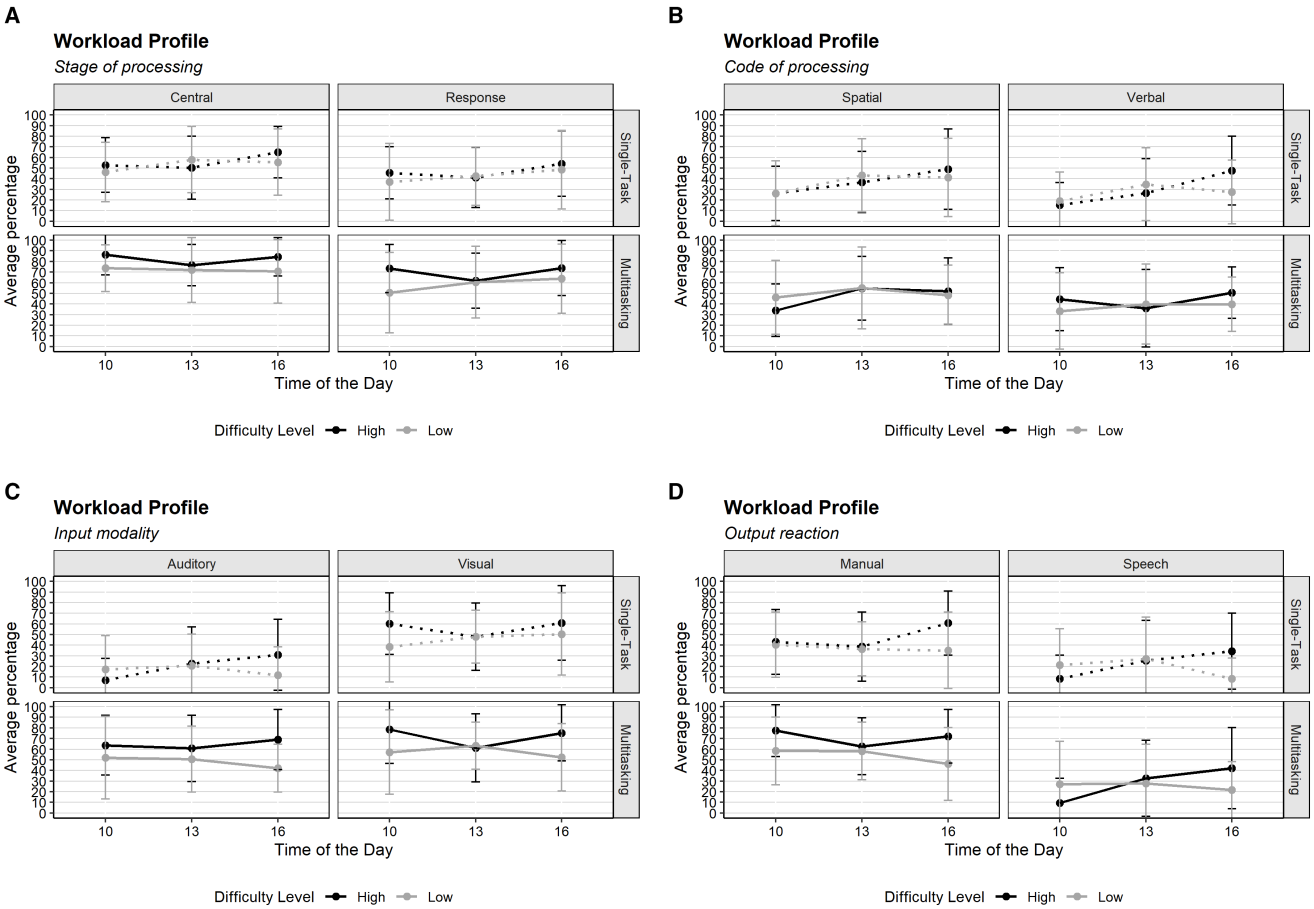
Resources of the Workload Profile by time of day, difficulty level, and task condition (the higher the percentage, the higher the use of that resource). **(A)** Stage of processing, **(B)** Code of processing, **(C)** Input modality, **(D)** Output reaction. Error bars represent the standard error of the mean.

We conducted a reliability analysis using Cronbach's alpha for the German version of the WP questionnaire, finding excellent internal consistency with α = 0.88 at T2 and α = 0.91 at T4. Item-level analyses revealed that the removal of any single item did not substantially improve reliability, with alpha values ranging from α = 0.88–0.90 when individual items were excluded. Corrected item-total correlations ranged from *r* = 0.70 (Speech) to *r* = 0.82 (Verbal), indicating strong relationships between each item and the overall scale. These values are comparable to those reported for the original English version of the WP, indicating that the German translation maintains a similar level of reliability. Figure 8 highlights the average percentage of resources used, showing a notable difference in the auditory resource due to the lack of communication task in the single task. Interestingly, although there was no speaking task, participants still perceived that they used this resource.

To examine the effects of multitasking and task difficulty on perceived workload, we employed LMMs for each resource dimension of the WP with *task condition, difficulty, group per hour* and *assessment point (T2, T4)* as fixed effects, and participant as a random intercept (conditional *R*^2^ = 0.57–0.85, marginal *R*^2^ = 0.08–0.33). We found a significant main effect of task condition across all resource (*p* < 0.001), with higher WP scores in the multitasking condition compared to the single-task condition. This result supports **Hypothesis 3**: participants allocated more cognitive resources when engaged in multitasking.

Regarding the effect of difficulty, significant main effects were found for Visual [*F*(1, 90) = 4.85, *p* = 0.03] and Manual [*F*(1, 90) = 6.11, *p* = 0.01] resources, where WP scores were higher under high difficulty conditions. However, no significant effects were observed for the other resource categories, partially supporting **Hypothesis 4**: visual and manual resources are higher when the task is difficult.

The group per hour did not yield significant main effects or interactions with difficulty or task condition across any resource category, suggesting that the allocation of cognitive resources remained stable throughout the day. Besides, no significant two-way or three-way interactions were found, indicating that the combined effects of difficulty, task condition, and group per hour did not produce meaningful variations in WP scores. Finally, the lack of a significant effect for trial suggests the absence of order-related effects.

### 4.3 Flow experience

**Figure 9:**
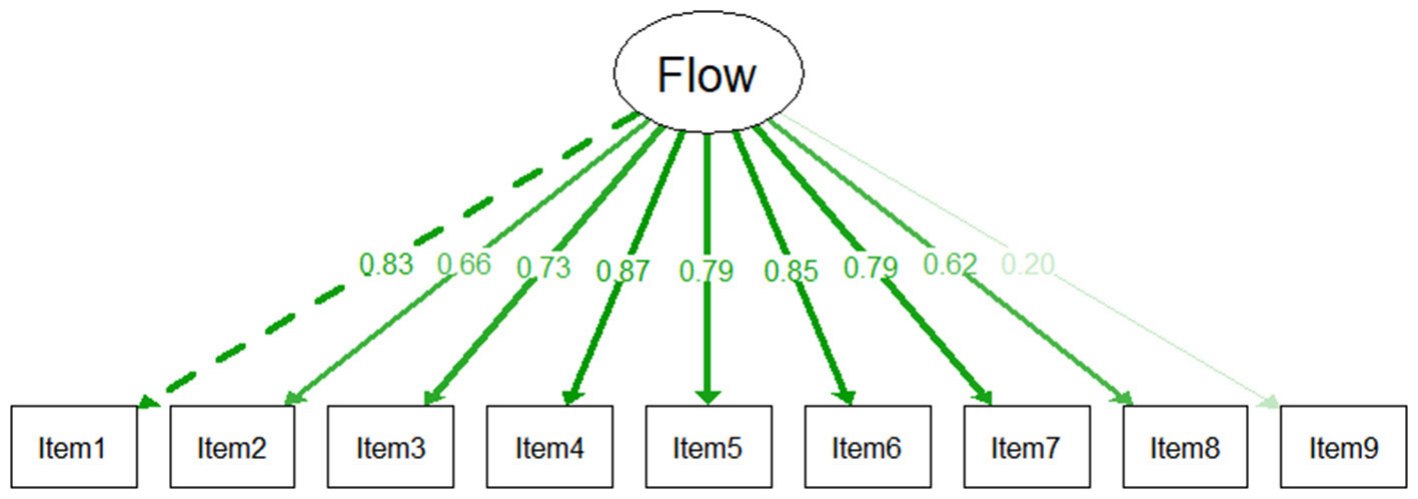
Path Diagram for the Flow-Experience scale.

We conducted a confirmatory factor analysis (CFA) to examine the unidimensional structure of the Flow Experience Questionnaire using items 1 to 9, as defined by the authors (Bartzik et al., 2021a). The one-factor model showed good fit to the data (CFI = 0.996, TLI = 0.995, RMSEA = 0.067, SRMR = 0.055). Standardized factor loadings were statistically significant for all items (*p* < 0.01). As presented in Figure 9, eight items showed strong loadings ranging, while Item 9 (in German: “*Bezogen auf die gerade ausgeführte Tätigkeit, wie sehr trifft es zu, dass … Sie im Tun an nichts anderes dachten*”) exhibited a weaker loading (λ = 0.20), consistent with the lower item discrimination and the negative skew (ceiling effect) observed in the item-level analyses (provided in the Supplementary Appendix C).

Despite this weaker performance, *Item 9* was retained because the overall scale remained psychometrically sound and the item's presence did not substantially reduce internal consistency. Furthermore, retaining the item ensured consistency with the original validated instrument and maintained comparability with established scoring protocols. Reliability analysis confirmed high internal consistency for the total scale, with Cronbach's α = 0.86 at T2 and α = 0.90 at T4, with a mean inter-item correlation of 0.41 at T2 and 0.49 at T4.

**Figure 10:**
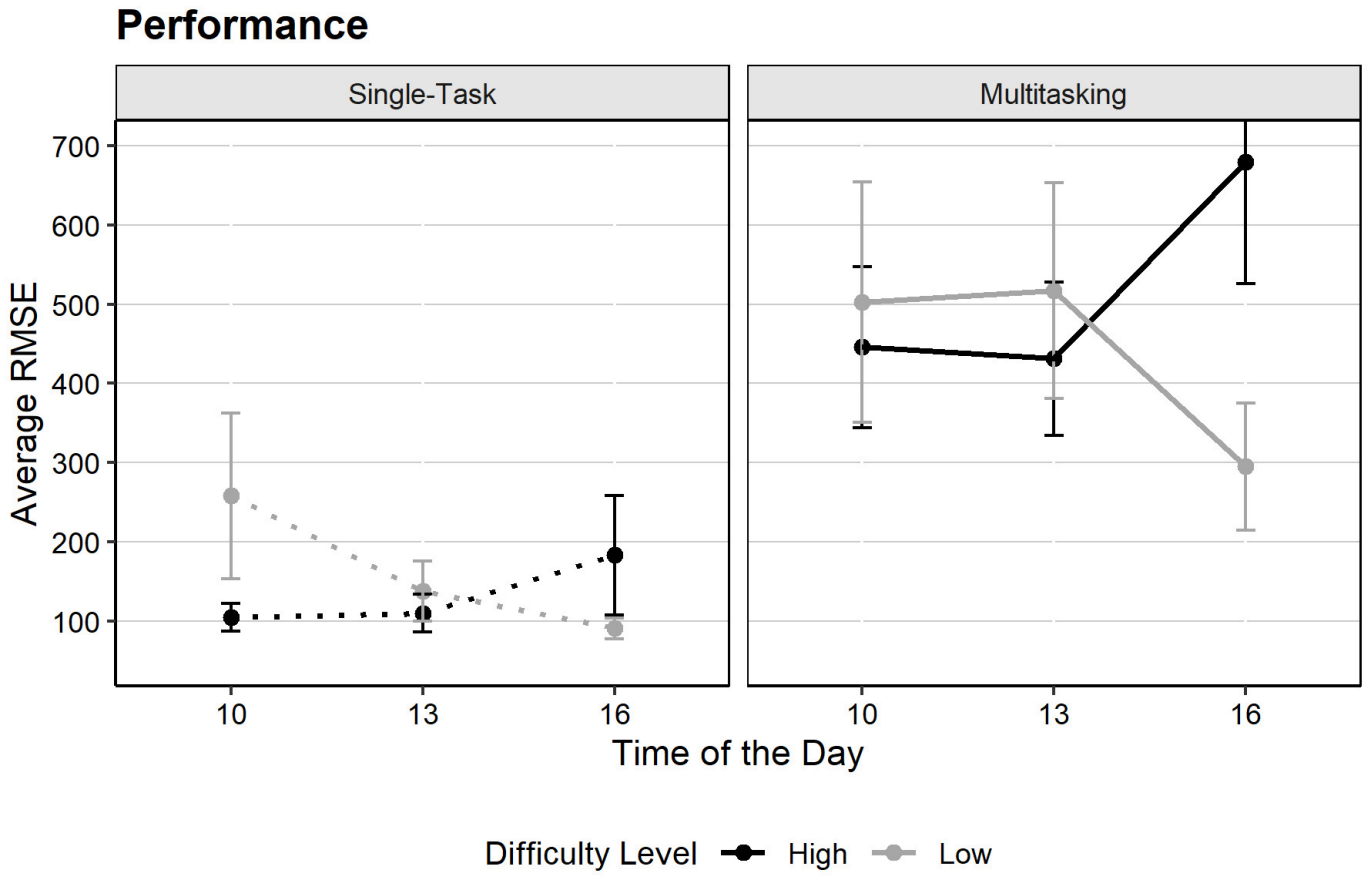
Mean Flow scores by time of day, difficulty level, and task condition (the higher the score, the greater the concentration). Error bars represent the standard error of the mean.

Figure 10 shows that participants on average had moderate to high flow. We performed LMM with *task condition, difficulty, group per hour* and *assessment point (T2, T4)* as fixed effects, and participant as a random intercept (conditional *R*^2^ = 0.29, marginal *R*^2^ = 0.10) to examinate the effects of task load on flow experience. The results revealed a significant main effect of task condition [*F*(1,89) = 14.38, *p* < 0.001, η*?*^2^ = 0.14], supporting **Hypothesis 5**: flow experience was significantly lower during multitasking (*M* = 3.56, SD = 1.01) compared to single-tasking (*M* = 4.05, SD = 1.01).

While the main effect of difficulty level was not significant, a significant interaction between difficulty and task condition emerged [*F*(1,89) = 4.21, *p* =0.043, η*?*^2^ = 0.05]. *Post-hoc* simple effects indicated that multitasking under high difficulty (*M* = 3.33, SD = 0.94) significantly reduced flow compared to the high-difficulty single-task (*M* = 4.08, SD = 0.97, *p* < 0.001). However, at low difficulty, the difference between task conditions was not significant. Consequently, we partially support **Hypothesis 6:** as high difficulty hindered flow specifically when participants were required to multitask. These results suggest that multitasking is particularly detrimental to the flow state when cognitive demands are high.

### 4.4 Performance

Our last two hypotheses examine the relationship between performance and task load using the ResMan task in MATB. We performed LMM with *task condition, difficulty, group per hour* and *trial* (1, 2) as fixed effects, and participant as a random intercept (conditional *R*^2^ = 0.56, marginal *R*^2^ = 0.21).

**Figure 11:**
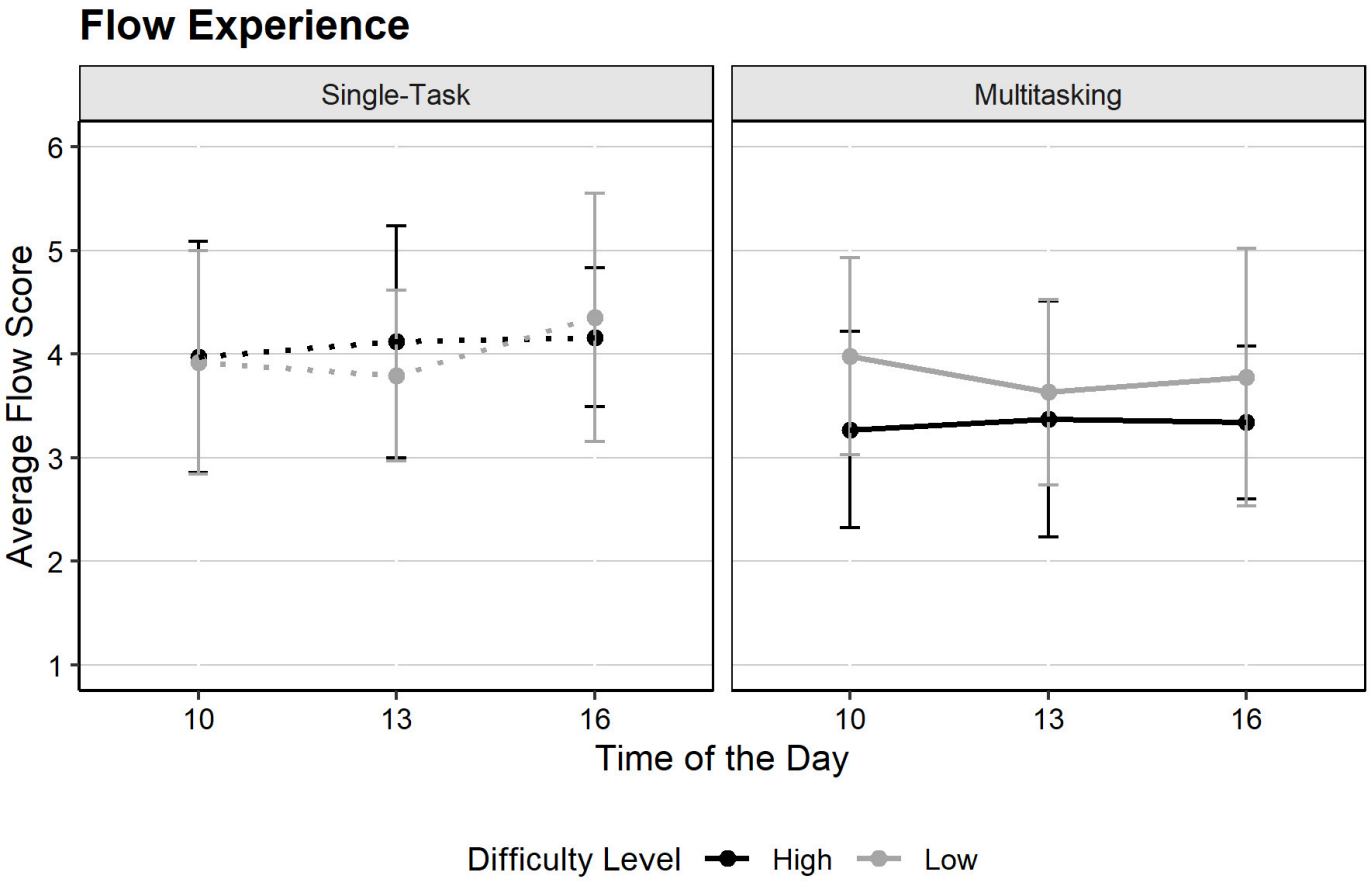
Average Performance scores by time of day, difficulty, and task condition (the higher the score, the lower the performance). Error bars represent the standard error of the mean.

The results showed a significant main effect of task condition [*F*(1, 89) = 64.96, *p* < 0.001, $\eta_{p}^{2}$ = 0.42], supporting our **Hypothesis 7**: multitasking significantly reduces performance compared to single-task conditions. Figure 11 clearly illustrates this difference, with multitasking having negative consequences on the scores, (considering that higher scores represent lower performance). Besides, a significant effect of trial was also observed [*F*(1, 89) = 8.11, *p* =0.005, $\eta_{p}^{2}$ = 0.08) indicating performance changes across successive trials, likely due to an order effect.

There were no significant main effects for group per hour, difficulty level, or their interactions. However, *post hoc* comparisons revealed significant performance differences between single-task and multitasking conditions under both high [*t*(89) = −6.65, *p* < 0.001] and low difficulty [*t*(89) = −4.75, *p* < 0.001]. Additional comparisons revealed a significant difference within the 4 p.m. multitasking condition [*t*(151) = 2.87, *p* = 0.019], where performance was significantly lower in the high difficulty level (*M* = 680.0, SD = 616.5) compared to the low difficulty condition (*M* = 294.81, SD = 321.4). While difficulty did not significantly affect performance overall, it did have an impact in specific situations, suggesting that our **Hypothesis 8** is partially supported: performance was lower when multitasking under high difficulty specifically in the late afternoon.

### 4.5 Correlations

Table 3 shows the correlation matrix between performance and all self-report constructs (Activation, Tension, Flow, and the eight Workload Profile resources). We observed three main patterns. First, Flow is strongly and positively associated with Activation (*r* = 0.55, *p* < 0.001) and negatively with Tension (*r* = −0.34, *p* < 0.001). This aligns with the theory that flow is a state of high energetic arousal but low tense arousal (Thayer, 1989). Second, all eight resources of the WP are highly inter-correlated (*r* = 0.37 to *r* = 0.78). This suggests that while the WP identifies distinct cognitive resources, they collectively tap into a general global workload. And third, Performance has positive correlations with Tension (*r* = 0.25, *p* = 0.016) and nearly all Workload dimensions (excluding Response and Speech). Since higher scores represent lower performance, these positive correlations indicate that as workload and tension increase, performance decreases.

**Table 3:** Correlation Matrix between performance and all self-report constructs.

Variable	1	2	3	4	5	6	7	8	9	10	11	12
1 Performance	–											
2 Activation	−0.10	–										
3 Tension	**0.25** ^*^	**-0.30** ^**^	–									
4 Flow	**-0.24** ^*^	**0.55** ^***^	**-0.34** ^***^	–								
5 Central	**0.35** ^***^	−0.06	0.18	−0.02	–							
6 Response	0.20	−0.04	0.07	0.06	**0.51** ^***^	–						
7 Spatial	**0.24** ^*^	−0.00	0.14	0.04	**0.52** ^***^	**0.56** ^***^	–					
8 Verbal	**0.26** ^*^	0.13	**0.21** ^*^	0.08	**0.52** ^***^	**0.37** ^***^	**0.61** ^***^	–				
9 Visual	**0.38** ^***^	−0.19	0.13	−0.13	**0.72** ^***^	**0.53** ^***^	**0.43** ^***^	**0.26** ^*^	–			
10 Auditory	**0.32** ^**^	−0.13	**0.22** ^*^	−0.08	**0.63** ^***^	**0.45** ^***^	**0.59** ^***^	**0.50** ^***^	**0.66** ^***^	–		
11 Manual	**0.32** ^**^	−0.10	0.12	−0.01	**0.75** ^***^	**0.52** ^***^	**0.60** ^***^	**0.44** ^***^	**0.78** ^***^	**0.73** ^***^	–	
12 Speech	0.11	0.07	0.12	0.02	**0.54** ^***^	**0.43** ^***^	**0.62** ^***^	**0.77** ^***^	0.20	**0.44** ^***^	**0.46** ^***^	–

*n* = 96, values in bold are significant:

^*^p < 0.05,

^**^p < 0.01,

^***^p < 0.001.

Regarding convergent validity, the strong correlation between Flow and Activation (*r* = 0.55, *p* < 0.001) supports the validity of the Flow scale as a measure of positive engagement. Additionally, Performance significantly correlates with Visual (*r* = 0.38), Central (*r* = 0.35), Manual (*r* = 0.32), and Auditory (*r* = 0.32) resources, confirming that the WP questionnaire validly captures the cognitive demands that interfere with task execution. Regarding discriminant validity, Flow shows almost zero correlation with all WP dimensions (all *p* > 0.05), providing strong evidence that the subjective experience of flow is a distinct psychological state from the perceived exertion of cognitive effort. Furthermore, the moderate negative correlation between Activation and Tension (*r* = −0.30, *p* = 0.003) confirms these are distinct constructs within the AD–ACL, one measuring energetic arousal and the other tense arousal.

## 5 Discussion

This study translated the Activation–Deactivation Adjective Check List (AD–ACL; Thayer, 1986) and the Workload Profile (WP; Tsang and Velazquez, 1996) into German, and validated these instruments, along with the Flow Experience questionnaire (Bartzik et al., 2021a), for assessing user states in control room settings. We demonstrated their psychometric robustness and sensitivity to task conditions (multitasking, single-task) and difficulty levels (high, low), as well as circadian effects across three groups (10:00 h, 13:00 h, 16:00 h) in a simulated scenario using the MATB software (Cegarra et al., 2020; Comstock et al., 1992; Santiago-Espada et al., 2011).

We examined how tasks load (both condition and difficulty) and group of the day influenced activation, tension, mental workload and flow experience together with performance on MATB. Table 4 presents a summary of these effects. Our findings reveal a complex interplay between these factors: while multitasking primarily drove declines in user states and performance, task difficulty emerged as a critical factor specifically during the late afternoon. These results provide a validated toolkit for assessing operator states in complex Human-Computer Interaction tasks.

**Table 4:** Summary of the task load effects.

Outcome measurement	Effect of task condition (type of task)	Effect of difficulty level	Effect of circadian rhythm
Activation	Increased under multitasking	Interacts with time and task condition (not main effect alone)	No main effect, but triple interaction effects: at 13:00 (increased during difficult multitask) and at 16:00 (increased during easy single-task)
Tension	Increased under multitasking, especially post-task	Increased under high difficulty, but only during multitasking	No effect observed
Mental Workload	Increased across all cognitive resources under multitasking	Increased under high difficulty, but only visual and manual resources	No effect observed
Flow experience	Decreased under multitasking	Decreased during multitasking only under high difficulty conditions	No effect observed
Performance	Decreased under multitasking	Partial effect, significant within multitasking at 16:00	No main effect, but specific impact at 16:00 under multitasking/high difficulty

### 5.1 Theoretical implications

Our findings on activation partially support our first hypothesis regarding circadian effect. While assessment point (T1, T2, T3, and T4) impacted activation levels, consistent effects for group per hour were not found. The significant main effect of the assessment point aligns with Thayer (1989) research showing fluctuations in daily activation levels. However, the lack of a main effect for group per hour suggests that circadian rhythms interacted with task complexity rather than following a simple linear trend. Compared to Thayer's original validation of the AD–ACL, our results suggest a more nuanced pattern influenced by contextual factors such as the laboratory setting, participants' anticipation, or motivational factors (e.g., compensation), which could elevate baseline arousal, particularly at T1.

Expanding the analysis, we found that multitasking resulted in higher energetic arousal than single-task, consistent with Matthews (2006); Matthews et al. (1990) findings. The interaction between difficulty level, group per hour, and task condition revealed that participants tested at 13:00 showed higher activation during difficult multitasking, while those at 16:00 exhibited higher activation in easier single-task conditions. This pattern aligns with Valdez (2019), who reported a post-lunch dip (14:00–16:00) and afternoon rebound (16:00–22:00). These results suggest that task load interacts with circadian rhythms to shape energetic arousal.

Regarding tension, our results strongly support our second hypothesis about task complexity effects on tension after task completion. Specifically, multitasking significantly increased tension levels, and difficulty effects emerged only under multitasking conditions, indicating that multitasking amplifies stress responses associated with task difficulty. These findings align with cognitive load theory, which posits that multitasking increases cognitive demands, potentially leading to heightened tension (Neerincx and De Greef, 1998). This aligns with Matthews (2006); Matthews et al. (1990), who found that task complexity significantly increases tension, which is more sensitive to stress-inducing conditions than alertness. Additionally, increased task difficulty requires greater concentration, causing tension after the activity is completed (Lu et al., 2022).

Compared to Thayer's original validation of the AD–ACL, our findings show that tense arousal is primarily influenced by task load rather than time-of-day. Thayer found that tense arousal varied significantly with task manipulations, particularly those that induced psychological stress, while circadian effects were less pronounced for tension than for energetic arousal. This convergence supports the validity of the German version of the AD–ACL in capturing task-induced tension. Furthermore, no significant circadian effects on tension were observed, suggesting that stress-related responses to multitasking and difficulty are independent of time-of-day influences, mirroring the original AD–ACL findings, reinforcing the notion that tension is more state-dependent than biologically regulated.

Turning now to the Workload Profile, results showed that multitasking consistently increased workload across all cognitive resources, fully supporting our third hypothesis. In contrast, task difficulty selectively impacted only visual and manual resources, offering partial support for the fourth hypothesis. These findings align with prior research indicating that demanding tasks require greater cognitive resources, increasing mental workload and reducing availability for concurrent tasks (Tsang and Wickens, 1988). When tasks compete for the same resources, the resulting strain can elevate workload, potentially causing cognitive overload and diminished allocation efficiency.

Our findings also corroborate Valéry et al. (2019), who emphasized that increased task difficulty in multitasking environments significantly alters resource allocation. Interestingly, speech resource utilization occurred despite the absence of a speaking task, consistent with Tsang et al. (1985), who suggested that speech-related cognitive resources engage, even without overt articulation, as a compensatory mechanism through inner speech or implicit verbalization. Likewise, Nedergaard et al. (2023) highlighted that task-relevant inner speech enhances attention and processing efficiency, suggesting that individuals may rely on inner speech to maintain performance even without explicit speaking. Although no significant main effect of time of day on mental workload was observed, the increased workload under multitasking and high difficulty is consistent with May and Hasher (2023) suggestion that task complexity is a primary driver of mental workload, with circadian effects potentially being subtle or context-dependent.

With respect to the flow experience, multitasking significantly reduced flow compared to single-tasking, fully supporting our fifth hypothesis. This aligns with previous findings showing that multitasking disrupts the flow state by fragmenting attention and increasing cognitive load (Peifer and Zipp, 2019; Pluut et al., 2024). Although the main effect of task difficulty was not significant, a significant interaction between difficulty and task condition emerged. *Post-hoc* analyses indicated that high difficulty reduced flow primarily when combined with multitasking, with no significant differences between difficulty levels in the single-task condition. This partially supports our sixth hypothesis, suggesting that difficulty affects flow only under multitasking demands, which aligns in part with prior studies that have emphasized that both too low and too high difficulty levels can hinder flow (Peifer and Zipp, 2019). Furthermore, no circadian effects on flow were detected, reinforcing that flow is primarily shaped by attentional demands rather than time-of-day variables.

Finally, task conditions significantly impacted performance, with multitasking leading to lower outcomes, supporting our seventh hypothesis. This underscores the cognitive burden of multitasking environments, as individuals split their attention across many tasks, reducing performance (Wickens et al., 2022). Performance was significantly lower during multitasking compared to single-tasking, both in low and high difficulty conditions; however, this was more pronounced at high difficulty level, indicating that task complexity amplifies multitasking challenges. Specifically, difficulty level significantly influenced performance at 4 p.m. under multitasking conditions, contrasting with (Adler and Benbunan-Fich 2015) who found better performance during low-difficulty multitasking.

In our study, difficulty level did not impact all conditions equally but became a critical factor later in the day, partially supporting our eighth hypothesis. The observed decline in performance at this hour suggests a possible accumulation of cognitive fatigue across the day (Van Dongen and Dinges, 2000), which may render demanding tasks particularly detrimental in multitasking environments (Hinss et al., 2022). This is in line with research indicating that time-of-day factors, individual chronotype, and circadian rhythms can influence task performance, particularly for effortful or attention-demanding tasks (May and Hasher, 2023; Schmidt et al., 2007). Additionally, complex tasks, especially those involving multitasking or problem-solving, are more influenced by circadian rhythms than simpler ones, with performance peaking when tasks align with an individual's chronotype and increasing difficulty shifting the optimal performance window earlier in the day (Wiłkość-Debczyńska and Liberacka-Dwojak, 2023). Though our study found limited direct effects of time of day, these findings highlight the relevance of considering temporal factors in performance research, especially in relation to task load and executive functioning.

### 5.2 Practical implications

These results have practical implications for the design of work environments in control rooms that account for the dynamic nature of dispatcher tasks and their cognitive demands throughout the day. As shown, activation, tension, mental workload, flow experience and task performance are not solely shaped by task condition or difficulty level, but in some cases by their interaction and time of day. Even simple tasks can negatively impact dispatchers by failing to engage cognitive resources effectively, resulting in a lack of stimulation (Howard et al., 2020; Jaquess et al., 2018). This complexity underlines the potential value of implementing adaptive assistance systems (AAS), which can automatically adjust system behavior based on the user and the context to prevent cognitive overload (Buchholz and Kopp, 2023; Witte et al., 2020). To design such systems effectively, it is essential to monitor user states which influence how support should be delivered (Schwarz et al., 2014). The validated German-language versions of the WP, AD–ACL, and Flow Experience questionnaires from this study provide practical tools for assessing these states in human-computer interaction with German-speaking users.

### 5.3 Limitations and future research

Although our study validates three questionnaires and uses them to show the effects of different combinations of task load on mental strain, it has some limitations. First, participants were volunteers, not professional control room operators, who participated in a simulated scenario, which may limit the generalizability of the findings. Nevertheless, the translated questionnaires have been already tested with dispatchers in real operational settings (Rey-Becerra et al., 2025). In this study, we found that dispatchers had moderate workload demands with high variability, stable tension and activation levels, moderate concentration, and positive emotional states during normal shifts without any critical situation.

Second, participants were from the local population, aged between 18 and 65, with a balanced gender distribution (50%-50%), whom we asked about their consumption of caffeine, theine, and nicotine. However, these aspects were not included in our analysis due to space constraints. For future studies, it is important to analyze possible differences based on age or gender, which could influence users' perception of their states, as well as possible effects on circadian rhythms.

Third, we noticed a potential ceiling effect in the flow scale, specifically regarding deep concentration (item 9), which likely limited the sensitivity to detect differences between high and maximal flow levels. Nevertheless, we retained this item to maintain comparability with established scoring protocols and because its inclusion did not substantially reduce the scale's internal consistency.

Four, we observed a significant order effect in task performance, suggesting that participants experienced a learning effect to the MATB interface across successive trials. However, because the effect size of the multitasking condition was more than five times larger than the order effect, the structural demand of the task remained the primary driver of performance degradation. While this learning curve is a common factor in complex simulations, future studies could employ more extensive pre-experimental training sessions to ensure performance reaches a stable plateau before data collection begins.

Finally, we adapted two questionnaires with different response scale: we replaced the classification symbols with a numerical scale in the AD–ACL, and we change the scale to a 0–100% in the WP. While this does not compromise validity (Eutsler and Lang, 2022), it may subtly influence response patterns. Future research should examine how demographics and stimulant use interact with mental strain and include professionals from relevant sectors to enhance validity.

## Data Availability

The datasets generated and analyzed during this study are not publicly available, as participants were informed and consented that their data would be used exclusively for the purposes of this study and would not be shared publicly. Requests to access the datasets should be directed to the corresponding author and will be considered on a case-by-case basis.

## Footnotes

1 For instance, technical malfunctions, infrastructure failures, extreme weather events, and cybersecurity threats—any of which could trigger cascading outages.

2 This framework provides a crucial distinction that originated in German terminology, where Rohmert and Rutenfranz (1975) clearly differentiated between “load” (Belastung) and “strain” (Beanspruchung; Schönpflug, 1987, as cited in Richter, 2000). According to the German standard DIN ISO 10075-1 (2017), mental workload corresponds to “psychische Arbeitsbelastung,” mental stress to “psychische Belastung,” and mental strain to “psychische Beanspruchung.”

3 The energetical state, as described by Gaillard (1993), is influenced by multiple factors: autonomic mechanisms like circadian rhythms, environmental factors (both external and internal), task anticipation, voluntary mental effort mobilization, and emotional responses. These factors create a dynamic interplay between attention and activation.

4 Both Bonferroni and Holm methods strongly control FWER under arbitrary dependence among tests (Calonico and Galiani, 2025), but Holm's procedure is uniformly more powerful, guaran-teed to never reject fewer hypotheses than traditional Bonferroni adjustment while maintaining identical FWER protection (Eichstaedt et al., 2013).
