# Supplementary material for: German version of subjective methods for measuring activation, mental workload, and attention in control rooms
Source: Front Psychol. 2026 Apr 7;17:1698078. doi: 10.3389/fpsyg.2026.1698078 (PMC13095825; doi:10.3389/fpsyg.2026.1698078)
Supplement: Supplementary file 1 [file Supplementary_file_1.docx]

Supplementary Material

# Appendix A

The Multi-Attribute Task Battery (MATB), created by Comstock et al. (1992), and adapted by Santiago-Espada et al. (2011) and Cegarra et al. (2020), is a computer-based task battery that has been released as an open-source platform for conducting experiments on human-automation interaction. It provides a robust way to manipulate mental stress (Albuquerque et al., 2020, Backs et al., 2005) to affect mental strain (Fairclough et al., 2005, Rodriguez Paras et al., 2016, Voorheis et al., 2005) and physiological parameters (Cerruti et al., 2010, Estepp et al., 2010, Fan et al., 2022, Miyake et al., 2009, Wilson and Russell, 2003, Wilson et al., 2010, Zhang et al., 2021), as well as to investigate the effects of automation (Bailey et al., 2006) or multitasking performance (Liu et al., 2016).

The MATB-II simulates four tasks that pilots perform during flight: (1) system monitoring (SysMon), (2) tracking (Track), (3) communication (Comm), and (4) resource management (ResMan). These tasks are presented on a computer screen and must be performed simultaneously. As the MATB-II is an open-source software, it was customized to include specific tasks and adjust difficulty levels. Hence, we selected three tasks—SysMon, Comm, and ResMan as they are particularly relevant to the role of dispatchers in the gas and electricity grid. We explain each of them as follow:

- SysMon: This task requires participants to monitor four indicators that fluctuate around the midpoint of a scale and respond appropriately to two colored alarm lights when they appear. The system records whether participants correctly responded to or ignored the alarms. This task simulates real-world scenarios in which dispatchers must detect deviations from normal conditions and respond to alerts.
- Comm: Participants must select a radio station and adjust the frequency in response to short audio prompts in English, ensuring the auditory information matches the visually displayed information on a panel. The system records whether the frequency was set correctly. This task replicates situations in which dispatchers receive phone calls and must determine whether the information is relevant to their work, requiring focused auditory attention.
- ResMan: In this task, participants regulate the fill level of a tank by turning pumps on or off according to given requirements. The system records the pump's state (ON/OFF) and the tank level at 30-second intervals. This task simulates the responsibility of dispatchers in maintaining optimal system conditions by monitoring and managing the gas or electricity grid to prevent disruptions.

# References

ALBUQUERQUE, I., TIWARI, A., PARENT, M., CASSANI, R., GAGNON, J.-F., LAFOND, D., TREMBLAY, S. & FALK, T. H. 2020. WAUC: A Multi-Modal Database for Mental Workload Assessment Under Physical Activity. *Frontiers in Neuroscience,* 14. <https://doi.org/10.3389/fnins.2020.549524>

BACKS, R. W., ROHDY, J. & BARNARD, J. 2005. CARDIAC CONTROL DURING DUAL-TASK PERFORMANCE OF VISUAL OR AUDITORY MONITORING WITH VISUAL-MANUAL TRACKING. *PSYCHOLOGIA,* 48**,** 66-83. <https://doi.org/10.2117/psysoc.2005.66>

BAILEY, N. R., SCERBO, M. W., FREEMAN, F. G., MIKULKA, P. J. & SCOTT, L. A. 2006. Comparison of a Brain-Based Adaptive System and a Manual Adaptable System for Invoking Automation. *Human Factors,* 48**,** 693-709. <https://doi.org/10.1518/001872006779166280>

CEGARRA, J., VALERY, B., AVRIL, E., CALMETTES, C. & NAVARRO, J. 2020. OpenMATB: A Multi-Attribute Task Battery promoting task customization, software extensibility and experiment replicability. *Behav Res Methods,* 52**,** 1980-1990. <https://doi.org/10.3758/s13428-020-01364-w>

CERRUTI, L. M., ESTEPP, J. R., MILLER, W. D. & CHRISTENSEN, J. C. 2010. Transcranial Doppler Assessment of Workload Transition in a Complex Task. *Proceedings of the Human Factors and Ergonomics Society Annual Meeting,* 54**,** 244-248. <https://doi.org/10.1177/154193121005400313>

COMSTOCK, J., RAYMOND, J. & ARNEGARD, R. J. 1992. The multi-attribute task battery for human operator workload and strategic behavior research. *In:* 1.15:104174), N. (ed.). <https://ntrs.nasa.gov/citations/19920007912>

ESTEPP, J. R., MONNIN, J. W., CHRISTENSEN, J. C. & WILSON, G. F. 2010. Evaluation of a Dry Electrode System for Electroencephalography: Applications for Psychophysiological Cognitive Workload Assessment. *Proceedings of the Human Factors and Ergonomics Society Annual Meeting,* 54**,** 210-214. <https://doi.org/10.1177/154193121005400305>

FAIRCLOUGH, S. H., VENABLES, L. & TATTERSALL, A. 2005. The influence of task demand and learning on the psychophysiological response. *Int J Psychophysiol,* 56**,** 171-84. <https://doi.org/10.1016/j.ijpsycho.2004.11.003>

FAN, Y., LIANG, J., CAO, X., PANG, L. & ZHANG, J. 2022. Effects of Noise Exposure and Mental Workload on Physiological Responses during Task Execution. *Int J Environ Res Public Health,* 19. <https://doi.org/10.3390/ijerph191912434>

LIU, S., WADESON, A., KIM, N. Y. & NAM, C. S. 2016. Effects of Working Memory Capacity, Task Switching, and Task Difficulty on Multitasking Performance. *Proceedings of the Human Factors and Ergonomics Society Annual Meeting,* 60**,** 502-506. <https://doi.org/10.1177/1541931213601114>

MIYAKE, S., YAMADA, S., SHOJI, T., TAKAE, Y., KUGE, N. & YAMAMURA, T. 2009. Physiological responses to workload change. A test/retest examination. *Appl Ergon,* 40**,** 987-96. <https://doi.org/10.1016/j.apergo.2009.02.005>

RODRIGUEZ PARAS, C., YANG, S., TIPPEY, K. & FERRIS, T. K. 2016. Physiological Indicators of the Cognitive Redline. *Proceedings of the Human Factors and Ergonomics Society Annual Meeting,* 59**,** 637-641. <https://doi.org/10.1177/1541931215591139>

SANTIAGO-ESPADA, Y., MYER, R. R., LATORELLA, K. A. & COMSTOCK, J. R., JR. 2011. The Multi-Attribute Task Battery II (MATB-II) Software for Human Performance and Workload Research: A User's Guide. *In:* (L-20031) (ed.). <https://ntrs.nasa.gov/citations/20110014456>

VOORHEIS, C. M., MORONEY, W. F., BIERS, D. W. & EGGEMEIER, F. T. 2005. In Search of Workload Context Effects Using a Compensatory Tracking Task. *Proceedings of the Human Factors and Ergonomics Society Annual Meeting,* 49**,** 2095-2098. <https://doi.org/10.1177/154193120504902406>

WILSON, G. F. & RUSSELL, C. A. 2003. Real-Time Assessment of Mental Workload Using Psychophysiological Measures and Artificial Neural Networks. *Human Factors,* 45**,** 635-644. <https://doi.org/10.1518/hfes.45.4.635.27088>

WILSON, G. F., RUSSELL, C. A., MONNIN, J. W., ESTEPP, J. R. & CHRISTENSEN, J. C. 2010. How Does Day-to-Day Variability in Psychophysiological Data Affect Classifier Accuracy? *Proceedings of the Human Factors and Ergonomics Society Annual Meeting,* 54**,** 264-268. <https://doi.org/10.1177/154193121005400317>

ZHANG, J., CAO, X., WANG, X., PANG, L., LIANG, J. & ZHANG, L. 2021. Physiological responses to elevated carbon dioxide concentration and mental workload during performing MATB tasks. *Building and Environment,* 195. <https://doi.org/10.1016/j.buildenv.2021.107752>

# Appendix B

## AD-ACL* Fragebogen zu Aktivierung

**Activation-Deactivation Adjective CheckList*

Jedes der folgenden Adjektive beschreibt ein Gefühl oder eine Stimmung. Bitte geben Sie jeweils an, wie Sie sich jetzt in diesem Moment fühlen. Arbeiten Sie die Fragen bitte zügig durch. Die erste Reaktion ist immer die beste. Question auf einer Skala von "1= gar nicht" bis "5= voll und ganz" fühle ich mich...

|  | Adjektive | 1 | 2 | 3 | 4 | 5 |
| --- | --- | --- | --- | --- | --- | --- |
| 1 | tatkräftig |  |  |  |  |  |
| 2 | aktiv |  |  |  |  |  |
| 3 | müde |  |  |  |  |  |
| 4 | voller Elan |  |  |  |  |  |
| 5 | angestrengt |  |  |  |  |  |
| 6 | nervös |  |  |  |  |  |
| 7 | munter |  |  |  |  |  |
| 8 | kraftvoll |  |  |  |  |  |
| 9 | schläfrig |  |  |  |  |  |
| 10 | angespannt |  |  |  |  |  |
| 11 | entspannt |  |  |  |  |  |
| 12 | unruhig |  |  |  |  |  |
| 13 | hellwach |  |  |  |  |  |
| 14 | gelassen |  |  |  |  |  |
| 15 | dösig |  |  |  |  |  |
| 16 | gefasst |  |  |  |  |  |
| 17 | friedlich |  |  |  |  |  |
| 18 | verkrampft |  |  |  |  |  |
| 19 | lebhaft |  |  |  |  |  |
| 20 | ruhig |  |  |  |  |  |

## Workload Profile zu psychischer Beanspruchung

Für verschiedene Aufgaben und Umgebungen braucht man zur Ausführung verschiedene geistige Ressourcen. Tragen Sie bitte nachfolgend mit einer ganzzahligen Prozentzahl ein, wie stark die jeweilige geistige Ressource durch die Aufgabe in Anspruch genommen wurde. 0 % steht für „die Aufgabe stellte keinerlei Anforderung“ und 100 % für „maximalen Ressourceneinsatz“.

| **Ressource** | **Prozentzahl 0-100** |
| --- | --- |
| 1. Zentrale geistige Verarbeitung z. B. beim Wahrnehmen (Erkennen, Wiedererkennen und Identifizieren von Objekten), Erinnern, Problemlösen und der Entscheidungsfindung |  |
| 2. Antwort Auswahl und Ausführung z. B. die Wahl des richtigen Pedals zum Bremsen eines Fahrzeuges. |  |
| 3. Verarbeitung räumlicher Information z. B. beim Erkennen der Position und Entfernung einer Radfahrerin im Straßenverkehr. |  |
| 4. Verarbeitung verbalen Materials z. B. beim Lesen von Text. |  |
| 5. Informationsaufnahme durch Sehen z. B. beim Betrachten eines Videos |  |
| 6. Informationsaufnahme durch Hören wie z. B. Musik hören |  |
| 7. Manuelle Ausführung wie z. B. Klavierspielen |  |
| 8. Sprachliche Reaktion wie z. B. in einer Unterhaltung |  |

# Appendix C

## Item Analysis for the Flow-Experience Questionnaire (N=96)

| **Flow-Experience at T2** | | | | | | | |
| --- | --- | --- | --- | --- | --- | --- | --- |
| **Row** | **Missings** | **Mean** | **SD** | **Skew** | **Item Difficulty** | **Item Discrimination** | **α if deleted** |
| Flow_i1 | 0.00 % | 3.62 | 1.41 | 0.05 | 0.60 | 0.71 | 0.83 |
| Flow_i2 | 0.00 % | 3.7 | 1.48 | -0.22 | 0.62 | 0.63 | 0.84 |
| Flow_i3 | 0.00 % | 3.4 | 1.45 | 0.23 | 0.57 | 0.61 | 0.84 |
| Flow_i4 | 0.00 % | 3.36 | 1.45 | -0.01 | 0.56 | 0.73 | 0.83 |
| Flow_i5 | 0.00 % | 3.6 | 1.35 | 0.13 | 0.60 | 0.65 | 0.84 |
| Flow_i6 | 0.00 % | 3.27 | 1.38 | 0.26 | 0.55 | 0.72 | 0.83 |
| Flow_i7 | 0.00 % | 3.83 | 1.29 | -0.17 | 0.64 | 0.68 | 0.84 |
| Flow_i8 | 0.00 % | 4.2 | 1.4 | -0.36 | 0.70 | 0.53 | 0.85 |
| Flow_i9 | 0.00 % | 4.65 | 1.52 | -0.9 | 0.77 | 0.10 | 0.89 |
| Mean inter-item-correlation=0.412 · Cronbach's α=0.859 | | | | | | | |

| **Flow-Experience at T4** | | | | | | | |
| --- | --- | --- | --- | --- | --- | --- | --- |
| **Row** | **Missings** | **Mean** | **SD** | **Skew** | **Item Difficulty** | **Item Discrimination** | **α if deleted** |
| Flow_i1 | 0.00 % | 3.65 | 1.36 | 0.01 | 0.61 | 0.80 | 0.88 |
| Flow_i2 | 0.00 % | 3.86 | 1.53 | -0.09 | 0.64 | 0.64 | 0.89 |
| Flow_i3 | 0.00 % | 3.7 | 1.49 | -0.08 | 0.62 | 0.71 | 0.89 |
| Flow_i4 | 0.00 % | 3.58 | 1.46 | 0.04 | 0.60 | 0.81 | 0.88 |
| Flow_i5 | 0.00 % | 3.79 | 1.52 | -0.15 | 0.63 | 0.74 | 0.88 |
| Flow_i6 | 0.00 % | 3.52 | 1.54 | 0.23 | 0.59 | 0.79 | 0.88 |
| Flow_i7 | 0.00 % | 3.81 | 1.4 | -0.15 | 0.64 | 0.71 | 0.89 |
| Flow_i8 | 0.00 % | 4.32 | 1.53 | -0.49 | 0.72 | 0.56 | 0.90 |
| Flow_i9 | 0.00 % | 4.66 | 1.35 | -0.84 | 0.78 | 0.27 | 0.92 |
| Mean inter-item-correlation=0.497 · Cronbach's α=0.900 | | | | | | | |

## Distribution of Item Responses. (N=96)

|  |  | **Value of the scale** | | | | | |
| --- | --- | --- | --- | --- | --- | --- | --- |
| **Item** | **Trial** | **1 (Never)** | **2** | **3** | **4** | **5** | **6 (Always)** |
| Item 1 | T2 | 0.052 | 0.188 | 0.260 | 0.188 | 0.208 | 0.104 |
|  | T4 | 0.042 | 0.198 | 0.219 | 0.250 | 0.198 | 0.094 |
| Item 2 | T2 | 0.073 | 0.198 | 0.156 | 0.198 | 0.281 | 0.094 |
|  | T4 | 0.042 | 0.208 | 0.177 | 0.177 | 0.208 | 0.188 |
| Item 3 | T2 | 0.073 | 0.260 | 0.198 | 0.240 | 0.125 | 0.104 |
|  | T4 | 0.073 | 0.177 | 0.198 | 0.219 | 0.198 | 0.135 |
| Item 4 | T2 | 0.125 | 0.167 | 0.250 | 0.198 | 0.198 | 0.062 |
|  | T4 | 0.073 | 0.188 | 0.240 | 0.198 | 0.188 | 0.115 |
| Item 5 | T2 | 0.052 | 0.146 | 0.323 | 0.208 | 0.167 | 0.104 |
|  | T4 | 0.073 | 0.167 | 0.167 | 0.250 | 0.177 | 0.167 |
| Item 6 | T2 | 0.083 | 0.250 | 0.260 | 0.188 | 0.156 | 0.062 |
|  | T4 | 0.052 | 0.302 | 0.167 | 0.177 | 0.156 | 0.146 |
| Item 7 | T2 | 0.031 | 0.135 | 0.229 | 0.271 | 0.240 | 0.094 |
|  | T4 | 0.052 | 0.146 | 0.198 | 0.281 | 0.188 | 0.135 |
| Item 8 | T2 | 0.021 | 0.135 | 0.146 | 0.240 | 0.240 | 0.219 |
|  | T4 | 0.042 | 0.104 | 0.177 | 0.156 | 0.208 | 0.312 |
| Item 9 | T2 | 0.042 | 0.083 | 0.115 | 0.125 | 0.219 | 0.417 |
|  | T4 | 0.021 | 0.062 | 0.135 | 0.146 | 0.292 | 0.344 |
